# The Recurrent Urinary Tract Infection Symptom Scale: Development and validation of a patient‐reported outcome measure

**DOI:** 10.1002/bco2.222

**Published:** 2023-01-17

**Authors:** Abigail F. Newlands, Lindsey Roberts, Kayleigh Maxwell, Melissa Kramer, Jessica L. Price, Katherine A. Finlay

**Affiliations:** ^1^ School of Psychology and Clinical Language Sciences University of Reading Reading UK; ^2^ School of Psychology University of Buckingham Buckingham UK; ^3^ Department of Psychology, Faculty of Natural Sciences University of Stirling Stirling UK; ^4^ Live UTI Free Ltd Dublin Ireland

**Keywords:** chronic pain, lower urinary tract symptoms, patient‐centred care, patient experience, patient‐reported outcomes, recurrent urinary tract infection, urinary tract infection, women's health

## Abstract

**Objectives:**

This study aimed to develop and validate a tailored patient‐reported outcome measure (PROM) evaluating the patient experience of recurrent urinary tract infection (rUTI) symptom severity. This measure was designed to supplement clinical testing methods, allowing full assessment of the patient experience of rUTI symptom burden, while enhancing patient‐centred UTI management and monitoring.

**Subjects and Methods:**

The Recurrent Urinary Tract Infection Symptom Scale (RUTISS) was developed and validated using a three‐stage methodology, in accordance with gold‐standard recommendations. Firstly, a two‐round Delphi study was conducted to gain insights from 15 international expert clinicians working in rUTI, developing an initial pool of novel questionnaire items, assessing content validity and making item refinements. Next, two phases of one‐to‐one semi‐structured cognitive interviews were conducted with a diverse sample of 28 people experiencing rUTI to assess questionnaire comprehensiveness and comprehensibility, making refinements after each phase. Finally, a comprehensive pilot of the RUTISS was conducted with 240 people experiencing rUTI across 24 countries, providing data for psychometric testing and item reduction.

**Results:**

Exploratory factor analysis indicated a four‐factor structure comprising: ‘urinary pain and discomfort’, ‘urinary urgency’, ‘bodily sensations’ and ‘urinary presentation’, together accounting for 75.4% of the total variance in data. Qualitative feedback from expert clinicians and patients indicated strong content validity for items, which was supported by high content validity indices in the Delphi study (I‐CVI > 0.75). Internal consistency and test–retest reliability of the RUTISS subscales were excellent (Cronbach's α = 0.87–0.94 and ICC = 0.73–0.82, respectively), and construct validity was strong (Spearman's ρ = 0.60–0.82).

**Conclusion:**

The RUTISS is a 28‐item questionnaire with excellent reliability and validity, which dynamically assesses patient‐reported rUTI symptoms and pain. This new PROM offers a unique opportunity to critically inform and strategically enhance the quality of rUTI management, patient‐clinician interactions, and shared‐decision making by monitoring key patient‐reported outcomes.

## 1 INTRODUCTION

Urinary tract infections (UTIs) affect more than 400 million people every year globally.^1^ As infections of the urethra, bladder, ureters and/or kidneys, UTIs cause symptoms including increased urination frequency and urgency, pain, and fever.^2^ Recurrent UTI (rUTI), defined by the European Association of Urology as experiencing two or more UTIs in 6 months or three or more in 1 year, is most common in females, where the risk of recurrence within a year of initial infection varies between 24% and 50%.^3^, ^4^ UTI episodes are associated with significant short‐term morbidity, on average causing approximately six symptomatic days of which two or more are significantly impacted, for example, in terms of ability to engage with work or study, social and sexual relationships.^5^ Characterised by repeated UTI experiences, and often by persistent lower urinary tract symptoms,^6^ rUTI is consistently found to be linked with lowered quality of life.^7^ Beyond the personal impact, UTIs also have significant socioeconomic consequences, especially in terms of healthcare costs where they represent 1%–3% of all primary care consultations and are the main indication in 13.7% of community‐based antibiotic prescriptions.^8^ The cost of antibiotic prescriptions for UTIs in primary care alone in 2019/20 was £47.6 million, a figure which has likely risen substantially given the increasing prevalence of UTIs and antibiotic‐resistant organisms.^8^, ^9^

Although established clinical approaches exist for UTI testing, microbiological research demonstrates that not all cases of significant positive bacteriuria indicate the existence of a UTI, and not all cases of a UTI present with positive bacteriuria that can be confirmed by culture.^10^, ^11^ It is not uncommon for patients to report symptoms of a UTI which are discrepant with standard clinical outcome measures,^10^, ^11^ yet currently, there is no way of assessing and validating the patient‐reported experience of UTI symptoms. Recent studies have recommended that rUTI clinicians go beyond guideline‐based management and incorporate a more patient‐centred perspective.^12^, ^13^ It is especially worthwhile, therefore, to consider the patient's own perspective in conjunction with any evaluation of clinical results. Indeed, there is a clear need for a validated patient‐reported outcome measure (PROM) that can efficiently incorporate the patient perspective in rUTI management, to aid in shared‐decision making and assess the effectiveness of rUTI treatment and interventions.^14^

Although PROMs have been validated for rUTI‐related conditions such as acute UTI (UTI Symptom Assessment, UTISA^15^) and acute cystitis (Acute Cystitis Symptom Score, ACSS^16^), there are no validated PROMs which specifically assess symptom severity in rUTI—a recurring condition, thus likely associated with a different, more enduring patient experience. The UTISA and ACSS do not evaluate frequency of UTI symptoms, nor do they extensively cover the breadth of symptoms experienced by the rUTI patient cohort.^2^, ^8^

**FIGURE 1:**
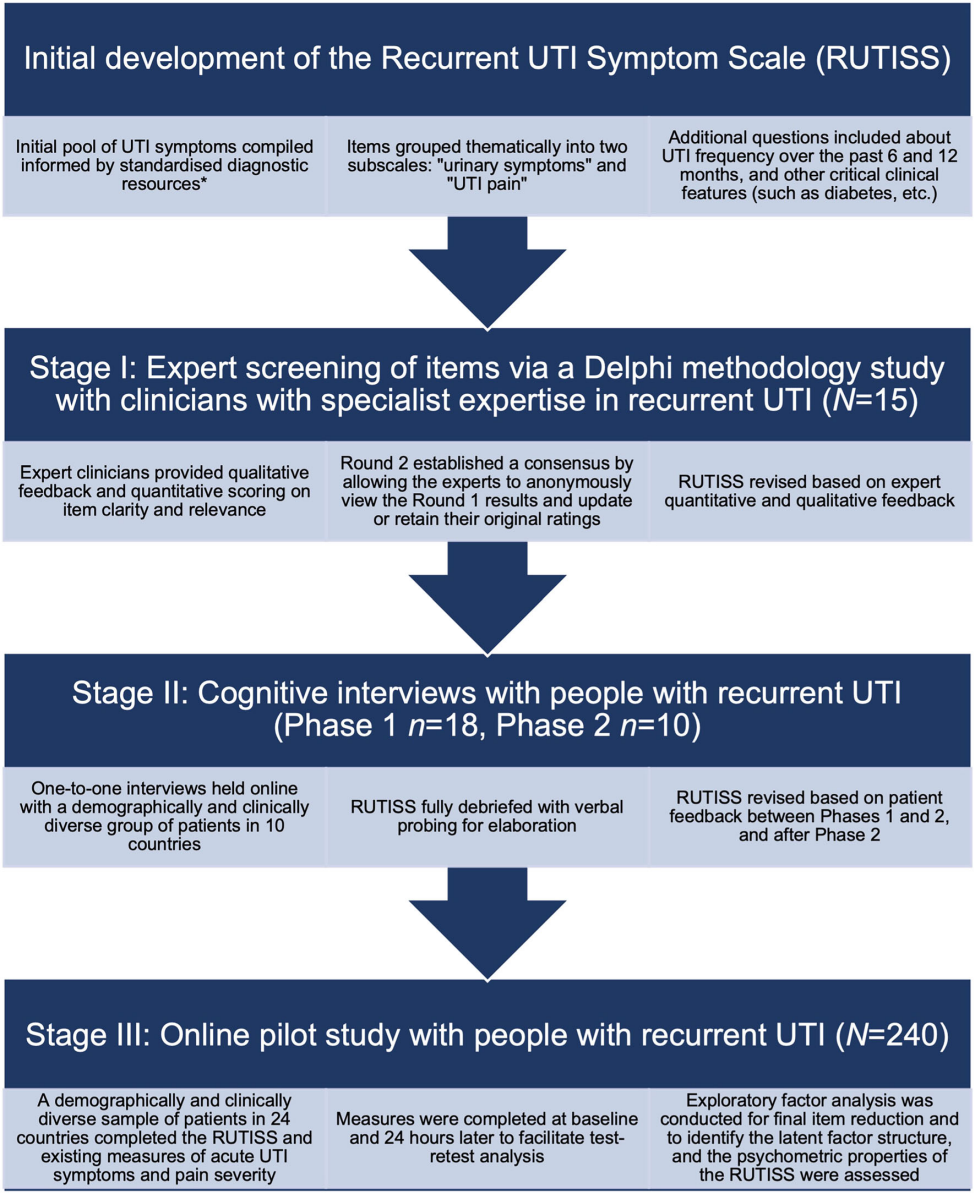
Three‐stage methodology used to develop and validate the Recurrent Urinary Tract Infection Symptom Scale. *Diagnostic resources including the NHS guidelines on UTIs^2^

This study aimed to develop and psychometrically validate the first rUTI‐specific PROM of symptom severity, in accordance with best practice recommendations by COnsensus‐based Standards for the selection of health Measurement INstruments (COSMIN; see Figure 1).^17^, ^18^

## 2 SUBJECTS AND METHODS

### 2.1 Initial development

To establish an initial dataset for question generation, a pool of PROM items was created following close inspection and consideration of diagnostic criteria and symptom presentation, resulting in two key subscales assessing severity of ‘urinary symptoms’ and ‘UTI pain’. To sensitively detect changes in pain and increase comprehensibility to participants, a widely‐used 11‐point numerical rating scale ranging from 0 (*no pain*) to 10 (*worst imaginable pain*) was initially implemented.^19^ A scoping exercise was conducted assessing other PROMs measuring comparable constructs (UTISA and ACSS^15^, ^16^) to establish the prospect of measure recall period, leading to the employment of a 24‐h period for this measure.

To ensure engagement with the UK Standards for Public Involvement, close consultation was maintained throughout this research with an international patient advocacy and research organisation with extensive interaction with people experiencing rUTI, Live UTI Free (https://liveutifree.com). The current project was pre‐registered at ClinicalTrials.gov (identifying no.: NCT05086900). Ethical approval was granted by the School of Psychology and Clinical Language Sciences Research Ethics Committee, University of Reading (project reference: 2021‐043‐KF).

### 2.2 Stage I: Expert screening

#### 2.2.1 Design

A two‐round Delphi methodology was employed to evaluate the content validity of the initial items as evaluated by expert clinician panellists (see Table S1 for all items tested at this stage).^20^ This technique applies a structured group‐interaction in which two or more rounds of questionnaire evaluation take place to effectively build a consensus.^20^ Round 1 sought to obtain the expert clinicians' views on the questionnaire items and instructions in terms of their relevance for rUTI and clarity. Round 2 built towards consensus by providing the expert clinicians with an opportunity to review the anonymised results from Round 1 and either update or retain their original feedback. The two rounds were spaced 28 days apart to minimise sample attrition.^20^ Consensus was reached in Round 2 and therefore a third round was not required.

#### 2.2.2 Participants and sampling

Thirty‐seven expert clinicians were invited to take part (*n* = 22 female, *n* = 15 male), of whom 15 were successfully recruited (*n* = 12 female, *n* = 3 male), meeting sample size recommendations for Delphi studies and COSMIN PROM development guidelines (see Table 1 for demographic characteristics).^17^, ^20^ Recruitment was purposive to achieve a heterogeneous sample with diverse perspectives from expert clinicians in primary and secondary care, reducing the risk of bias.^21^ Inclusion criteria included currently working as either a general health practitioner or specialist doctor/nurse practitioner within a relevant discipline (e.g., urology). A snowball sampling method was employed whereby an initial pool of expert clinicians was invited via email and encouraged to share the invitation with other potentially eligible participants. Equal proportions of primary and secondary care practitioners were recruited. Expert clinicians were aged between 32 and 64 years (*M* = 46.8, *SD* = 9.24) and experience in treating rUTI ranged from 2 to 30 years (*M* = 13.2, *SD* = 7.95). Eighty per cent retention was achieved in Round 2.

**TABLE 1:** Participant demographic characteristics

Characteristic	*n*	%
Expert clinicians		
Profession^a^		
General practitioner	8	53.3
Specialist doctor	6	40.0
Specialist nurse practitioner	1	6.67
Gender		
Female	12	80.0
Male	3	20.0
Country of practice		
United States	8	53.3
United Kingdom	6	40.0
Canada	1	6.67
Ethnicity		
White	10	66.7
Asian	4	26.7
Other	1	6.67
Cognitive interview patients		
Gender		
Female	25	89.3
Male	2	7.14
Non‐binary	1	3.57
Country of residence		
United States	9	32.1
United Kingdom	7	25.0
Canada	4	14.3
Australia	2	7.14
Austria	1	3.57
The Netherlands	1	3.57
New Zealand	1	3.57
South Africa	1	3.57
Ukraine	1	3.57
United Arab Emirates	1	3.57
Ethnicity		
White	25	89.3
Asian	2	7.14
Spanish or Latino American	1	3.57
Fluency in English		
Native or bilingual	25	89.3
Advanced or proficient	3	10.7
Relationship status		
Married or in a civil partnership	17	60.7
In a relationship	6	21.4
Single	4	14.3
Separated or divorced	1	3.57
Pilot patients		
Gender		
Female	233	97.1
Male	5	2.08
Non‐binary	2	0.83
Country of residence		
United States	93	38.8
United Kingdom	77	32.1
Australia	18	7.50
Canada	16	6.67
France	4	1.67
Sweden	4	1.67
Malaysia	3	1.25
New Zealand	3	1.25
Spain	3	1.25
Ireland	2	0.83
The Netherlands	2	0.83
Portugal	2	0.83
South Africa	2	0.83
Other^b^	11	4.58
Ethnicity		
White	214	89.2
Asian	11	4.58
Spanish or Latino American	6	2.50
Mixed	2	0.83
Black or African American	1	0.42
Native Hawaiian or other Pacific Islander	1	0.42
Prefer not to say	5	2.08
Fluency in English		
Native or bilingual	195	81.2
Advanced or proficient	45	18.8
Relationship status		
Married or in a civil partnership	123	51.3
In a relationship	78	32.5
Single	32	13.3
Engaged	4	1.67
Widowed	1	0.42
Prefer not to say	2	0.83

*Note*: Expert clinicians *N* = 15. Cognitive interview patients *N* = 28. Pilot patients *N* = 240 (*n* = 106 participants were retained to complete the Test–Retest Assessment). Patient participants identifying as non‐binary reported female biological sex.

^a^ Of the specialist clinicians, 71.4% (*n* = 5) worked in urology and 28.6% (*n* = 2) in urogynaecology.

^b^ Other countries of residence where *n* = 1 include Austria, Costa Rica, Germany, Greece, India, Kenya, the Philippines, Romania, Saint Vincent and the Grenadines, Switzerland and Ukraine.

#### 2.2.3 Procedure

Expert clinicians were presented with each preliminary item and instruction sequentially using an encrypted online survey tool (REDCap; https://www.project-redcap.org). In Round 1, participants were asked to rate each for relevance and clarity on a 7‐point scale (0 = *not at all relevant/clear*, 6 = *highly relevant/clear*) and to provide qualitative feedback to explain quantitative responses.^22^ After rating each subscale's components individually, expert clinicians reviewed the entire section in full, and provided final comments about the PROM's comprehensiveness and comprehensibility.^17^

In Round 2, each RUTISS item and instruction was presented alongside the median relevance and clarity ratings and anonymised qualitative feedback obtained in Round 1. Expert clinicians retained or updated their original ratings and provided further commentary.

#### 2.2.4 Data handling

Following Round 2, new median ratings were calculated and analysed in conjunction with the qualitative feedback. Content validity indices for items (I‐CVI) were computed by dividing the number of expert clinicians who scored an item's relevance/clarity as at least four out of six by the total number of expert clinicians.^22^ It was specified a priori that a minimum I‐CVI of 0.75 would be required to indicate acceptable consensus of content validity, with a minimum median score of 4 (achieved for all except four items; see Section 3).^20^, ^21^ Refinements were made to create a second version of the RUTISS, ready for patient testing.

### 2.3 Stage II: Patient cognitive interviews

#### 2.3.1 Design

One‐to‐one semi‐structured interviews with people with rUTI were then conducted online using Microsoft Teams. A cognitive debriefing technique was used, encouraging participants to think aloud as they reviewed and answered the PROM questions, in order to study the ways in which the questions may be mentally processed and where problems may arise (see Table S2 for all items tested at this stage).^23^ Interviews took place in two phases, facilitating an iterative approach in which the RUTISS was refined partway through with an opportunity to validate these refinements in Phase 2.^23^ All interviews were conducted by the first author to ensure homogeneity in interview style.^23^

#### 2.3.2 Participants and sampling

A clinically and demographically diverse sample of 28 adults experiencing rUTI was purposively recruited (see Table 1 for demographic characteristics and Figure S1 for sampling and recruitment strategy). Inclusion criteria comprised a minimum age of 18 years old, native or advanced fluency in English, and meeting the minimum diagnostic criteria for rUTI (≥2 UTIs in 6 months, or ≥3 UTIs in 12 months) based on self‐report.^3^, ^11^ Participants were excluded if they reported a current diagnosis of interstitial cystitis, used urinary catheterisation, or were pregnant. A sample size of approximately 30 participants for cognitive interview was sought to attain confidence that all possible problems with the PROM had been identified.^17^, ^24^ Seventy‐three potential participants completed the consent form and screening survey without being excluded, from which as diverse a sample as possible was selected using a maximum variation sampling strategy (*N* = 28).^25^

The final sample was aged between 18 and 82 years (*M* = 46.8, *SD* = 16.9). The median number of UTI episodes in the past 6 and 12 months was 4 (*IQR* = 4) and 7 (*IQR* = 8), respectively. Years of UTI symptoms ranged from 1 to 65 (*M* = 17.3, *SD* = 14.5) and years of UTI impact ranged from 1 to 60 (*M* = 8.9, *SD* = 12.0).

#### 2.3.3 Procedure

Participants provided written informed consent prior to their interview after reading a Participant Information Sheet detailing the study aims and ethical considerations. While displaying the RUTISS to participants, think‐aloud and verbal probing techniques were applied to identify the possible ways in which each instruction and item should be improved and to determine the questionnaire's overall comprehensiveness and comprehensibility.^17^, ^23^ The interviewer invited participants to think aloud their thought processes while deciding their answer to each RUTISS question. A topic guide (see Figure S2) was used, including relevant scripted probes to encourage elaboration and clarification.^23^ Spontaneous probes were employed where appropriate, and additional questions were asked about the scales and response options, the time taken to complete the questions, and the questionnaire's layout and formatting. Anonymised field notes were created during each interview and a written summary was formalised once each interview was complete, to build richness and facilitate transcript interpretation.^23^

#### 2.3.4 Data handling

Anonymised verbatim transcripts of the interviews were created using speech‐to‐text software (Otter; https://otter.ai) with transcription errors manually corrected. The corresponding portions from every interview transcript for each PROM item were collated to create individual documents for each item containing all feedback pertaining to that question from across the entire sample. Top‐down question feature coding was applied to these summaries using the Question Appraisal System (QAS‐99).^23^, ^26^ The systematic evaluation of every response to each PROM item was conducted to identify potential problems, which were corrected based on verbatim quotes and interviewer field notes through collaborative analysis with the entire research team.^23^, ^26^ If there were any uncertainties found in Phase 1 about whether to include or exclude an item, the item was retained in Phase 2 to gain further feedback. A third version of the RUTISS was created at this stage and used in the second phase of interviews. The same process was undertaken after Phase 2, with an amended version created for pilot testing.

### 2.4 Stage III: Pilot

#### 2.4.1 Design and hypotheses

A two‐part cross‐sectional survey was conducted online with rUTI patients to gather data for psychometric testing of the RUTISS and final item reduction. A within‐subjects design was used, in which participants completed the same procedure twice: (a) at baseline (Baseline Assessment) and (b) 24 h later to facilitate test–retest reliability analysis (Test–Retest Assessment). This 24‐h window was designed to capture two timepoints at which UTI symptoms may remain approximately similar^17^, ^27^ and is in accordance with the timeframes used by the UTISA and ACSS.^15^, ^16^

To facilitate construct validity analyses, participants also completed the UTISA and Numerical Pain Rating Scale (NPRS), existing validated instruments measuring urinary symptoms and pain.^15^, ^28^ It was hypothesised a priori that there would be moderate to strong correlations (Spearman's ρ > 0.50) between the RUTISS subscale scores and the UTISA and NPRS scores.^17^, ^18^ Given their conceptual similarities, it was also expected that the *urinary symptoms* subscale would correlate most strongly with the UTISA scores, and the *UTI pain* subscale most strongly with the NPRS scores.

It was hypothesised that the factor structure would differentiate between *urinary symptoms* and *UTI pain* as distinct concepts.^29^ It was finally hypothesised that the global rating of change (GRC) scale would statistically significantly negatively predict the RUTISS subscale scores (patient‐reported improvement over the past 24 h was expected to predict lower symptom and pain severity scores).

#### 2.4.2 Participants and sampling

A total of 240 adults meeting the diagnostic criteria for rUTI completed the Baseline Assessment and 106 (44.2%) were retained to complete the Test–Retest Assessment (see Table 1 for demographic characteristics and Figure S3 for sampling and recruitment strategy). The inclusion/exclusion criteria used in the cognitive interview stage were also adopted for this pilot. A minimum of 165 participants were sought to complete the Baseline Assessment, based on the minimum standard of five participants per questionnaire item for exploratory factor analysis (EFA; RUTISS = 33 items before final item reduction).^17^ Sampling adequacy was therefore exceeded in the final sample. Participants were aged between 18 and 84 years (*M =* 45.0, *SD =* 17.3).

#### 2.4.3 Procedure

After reviewing a Participant Information Sheet detailing the study aims and ethical issues, participants electronically provided consent. Eligible participants who were not excluded during an initial demographics questionnaire proceeded to complete the RUTISS, UTISA and NPRS. The UTISA is a 14‐item questionnaire assessing the ‘severity’ and ‘bothersomeness’ of seven acute UTI symptoms over the past 24 h.^15^ Only the 7‐item ‘severity’ subscale was utilised in this study due to its relevance to the RUTISS constructs. The NPRS is a 4‐item questionnaire assessing respondents' current level of pain intensity, and their lowest, highest, and average level of pain intensity in the past 24 h.^28^

Twenty‐four hours after completing the Baseline Assessment, participants were invited by email to complete the same questionnaires again (Test–Retest Assessment).

#### 2.4.4 Data handling

After screening the data for ineligible participants and missing data (see Figure S3), summed scores were calculated for each RUTISS subscale. EFA was conducted to determine the latent factor structure (structural validity) of the RUTISS and perform final item reduction.^17^, ^29^ The psychometric and predictive properties of the questionnaire were analysed (see Figure S4 for data handling strategy and assumptions testing).

## 3 RESULTS

### 3.1 Stage I: Expert screening

All except four items achieved I‐CVI for relevance and/or clarity greater than the minimum criterion of 0.75 and median ratings of at least 4. The four items with lower ratings were treated as follows: two items (‘cloudy urine’: I‐CVI for relevance = 0.42 and I‐CVI for clarity = 0.67; ‘change in temperature’: I‐CVI for relevance = 0.67) were retained for further exploration at the cognitive interview stage due to standardised inclusion in UTI patient advice guides^2^; the ‘menopausal symptoms’ item (I‐CVI for clarity = 0.67) was refined by soliciting expert qualitative feedback which provided supplementary examples of relevant symptoms; and the ‘constipation’ item (I‐CVI for relevance = 0.58 and I‐CVI for clarity = 0.67) was relocated to the critical clinical features section of the RUTISS rather than listed as a symptom.

The quantitative results were examined alongside the qualitative feedback to make necessary refinements to the PROM (see Table S1 for all items tested at this stage, including refinements and I‐CVI). Overall, updates were minor and reflected opportunities to enhance clarity by providing more detailed instructions and definitions.

### 3.2 Stage II: Patient cognitive interviews

Priorities for improvement in the second version of the RUTISS indicated that clarity and comprehensibility could be improved by defining the intended meaning using relevant examples and simplifying the language to enhance readability (see Table S2 for all items tested at this stage, including refinements). All recommended changes were reflected in a third version of the RUTISS and tested in Phase 2. Only minor refinements were made after this phase with no new items added, indicating data saturation was reached.^23^

### 3.3 Stage III: Pilot

#### 3.3.1 Descriptive statistics

Approximately half of participants (56.3%, *n =* 135) reported taking antibiotics at the time of participation, either to treat a current UTI, prevent new UTIs, and/or for other indications. Approximately three‐quarters (76.3%, *n =* 183) reported managing their rUTI with non‐antibiotic treatment including natural remedies or supplements. Approximately three‐quarters of participants (77.5%, *n =* 186) reported experiencing persistent lower urinary tract symptoms for at least the past 3 months, indicative of UTI recurrence,^6^ with the remainder of participants reporting symptoms which occur on an episodic basis. The mean number of episodes of symptoms reported in the past 6 months was 6.81 (*SD =* 24.3), and the mean in the past year was 13.9 (*SD =* 48.4).

Observed RUTISS subscale scores indicated the breadth in patient symptom experiences, with scores ranging from 0 to 69 for the *urinary symptoms* subscale (maximum possible = 70, *M =* 23.2, *SD =* 16.9) and from 0 to 92 for the *UTI pain* subscale (maximum possible = 100, *M =* 26.2, *SD =* 23.0). Similarly broad ranges were observed in the UTISA and NPRS data (UTISA range = 0–20, maximum possible = 21, *M =* 6.71, *SD =* 4.75; NPRS range = 0–37, maximum possible = 40, *M =* 12.1, *SD =* 10.0). Approximately two‐thirds (69.1%, *n =* 161) of female participants reported a UTI at the time of testing, as demonstrated by scoring greater than 3 on the UTISA, the cut‐off value for prediction of an uncomplicated UTI in females (*M =* 6.73, *SD =* 4.77).^27^ These participants reported statistically significantly greater *urinary symptom* scores (*M =* 39.1, *SD =* 22.2) and *UTI pain* scores (*M =* 34.3, *SD =* 22.4) in the RUTISS than participants who did not report a UTI at the time of testing (*urinary symptoms*: *M =* 11.3, *SD =* 11.6, *t*(231) = 10.0, *p* < 0.001; *UTI pain*: *M =* 7.93, *SD =* 10.6, *t*(231) = 9.60, *p* < 0.001). Most non‐female participants (71.4%, *n* = 5) scored greater than 3 on the UTISA (*M =* 6.14, *SD =* 4.53).

#### 3.3.2 Exploratory factor analysis

There were four instances of inter‐item correlation coefficients greater than 0.80: one in the *urinary symptoms* subscale (C1‐C2) and three in the *UTI pain* subscale (D1‐D4, D2‐D3, D2‐D6). It was identified through the patient cognitive interviews that C1 (increased urinary frequency) and C2 (increased urgency) were conceptually related, but that they represent clinically and personally important differences in terms of symptom burden. The strong correlations between the listed pain items were also expected, given that D1 and D4 both relate to pain while urinating, and D2, D3, and D6 to pain while not urinating. Therefore, these items were retained. Bartlett's Test of Sphericity was statistically significant (*p* < 0.001), confirming the absence of multicollinearity.^29^ The Kaiser–Meyer–Olkin Measure of Sampling Adequacy estimate was high at 0.92, demonstrating the suitability of the data for EFA.^29^

Items C3 (unintentionally leaking urine) and C7 (visible blood in the urine) did not load above 0.30 on any initial factors and showed extracted communalities of less than 0.40, therefore were removed.^29^ Items C10 (feeling generally unwell), C11 (fever), and C12 (chills) demonstrated problematic multiple cross‐loadings, therefore were removed.^29^ All other items met the minimum criteria for communalities and factor loadings.

The final four‐factor structure consists of the following factors: ‘urinary pain and discomfort’, ‘urinary urgency’, ‘bodily sensations’, and ‘urinary presentation’ (see Table 2). These factors represent a strong fit for the data and collectively account for 75.4% of the total variance in scores. The factor structure capably distinguishes between *urinary symptoms* and *UTI pain*. The final version of the RUTISS consists of 28 items (see Table 3; the full typeset questionnaire is available in Figure S5).

**TABLE 2:** Final four‐factor structure for the RUTISS

Factor: Item	Factor loading				Communality
	1	2	3	4	
Factor‐1: Urinary pain and discomfort					
D1. When you are urinating, how has your pain or discomfort been on average over the past 24 hours?	**0.59**	0.38	0.18	**0.52**	0.80
D2. When you are not urinating, how has your pain or discomfort been on average over the past 24 hours?	**0.84**	0.30	0.31	0.18	0.92
D3. What is your level of pain or discomfort right now?	**0.67**	0.33	0.33	0.22	0.72
D4. Pain or burning sensation when you are urinating.	**0.47**	0.39	0.10	**0.58**	0.72
D5. Pain or burning sensation within the 30 minutes after urinating.	**0.68**	0.27	0.22	**0.41**	0.76
D6. Pain or discomfort around the urethra when you are not urinating.	**0.81**	0.25	0.18	0.26	0.81
Factor‐2: Urinary urgency					
C1. Needing to urinate more frequently than normal.	0.21	**0.85**	0.22	0.20	0.84
C2. Needing to urinate more urgently or more suddenly than normal.	0.27	**0.78**	0.22	0.17	0.75
C4. Feeling as though you are unable to completely empty your bladder.	0.22	**0.51**	0.09	0.33	0.43
C5. Feeling as though you have the urge to urinate despite having just urinated.	0.36	**0.67**	0.14	0.28	0.67
Factor‐3: Bodily sensations					
D7. Pain or discomfort in your pelvis or lower tummy/abdomen (including bladder pressure).	**0.52**	0.31	**0.45**	0.22	0.62
D8. Pain or discomfort in your lower back.	0.16	0.15	**0.80**	0.13	0.70
D9. Pain or discomfort in your side/flank.	0.14	0.22	**0.76**	0.16	0.67
D10. Pain or discomfort radiating down your legs.	0.29	0.07	**0.65**	0.20	0.60
Factor‐4: Urinary presentation					
C6. Urine with an unusually strong or unpleasant smell.	0.16	0.18	0.18	**0.69**	0.57
C8. Cloudy urine.	0.26	0.28	0.24	**0.60**	0.57
C9. Debris or floating particles in your urine.	0.36	0.18	0.33	**0.45**	0.47

*Note*: *N =* 240. A four‐factor structure was identified through exploratory factor analysis, indicating the distinctive profiles of items assessing urinary symptoms and UTI pain. These four factors together accounted for 75.4% of the total variance in scores. The extraction method was Principal Axis Factoring with Kaiser‐Varimax rotation. Factor loadings above 0.40 are in bold.

**TABLE 3:** Final 28 items included in the Recurrent Urinary Tract Infection Symptom Scale

Section/item number	Instruction/item
Section A: Symptom frequency	The following questions are about how often you experience UTI symptoms. Please consider UTIs that may or may not have been medically diagnosed.
A1	Have you had UTI symptoms that feel continuous and do not fully subside **for at least the past 3 months** ?
A2	Approximately how many episodes of UTI symptoms have you had in the **past 6 months**?
A3	Approximately how many episodes of UTI symptoms have you had in the **past 12 months**?
Section B: Global rating of change	The following questions are about any **change** in your UTI symptoms.
B1	Please consider how you typically experience UTI symptoms. To what extent have your UTI symptoms over the PAST 24 HOURS been **better or worse than your typical experience** ?
Section C: Urinary symptoms	The following questions are about your **UTI symptoms other than pain or discomfort** . Please indicate whether you have experienced any of the following symptoms **related to UTI** in the PAST 24 HOURS, and if so, how SEVERE they were:
C1	Needing to urinate more frequently than normal.
C2	Needing to urinate more urgently or more suddenly than normal.
C3	Feeling as though you are unable to completely empty your bladder.
C4	Feeling as though you have the urge to urinate despite having just urinated.
C5	Urine with an unusually strong or unpleasant smell.
C6	Cloudy urine.
C7	Debris or floating particles in your urine.
Section D: UTI pain	The following questions are about any **pain or discomfort in your lower abdomen, genitals and/or bladder** , related to your UTI(s).
D1	When you are urinating, how has your pain or discomfort been **on average over the past 24 hours**?
D2	When you are not urinating, how has your pain or discomfort been **on average over the past 24 hours**?
D3	What is your level of pain or discomfort **right now**?
Instruction	**Please indicate whether you have experienced any of the following symptoms related to UTI in the PAST 24 HOURS, and if so, how SEVERE they were:**
D4	Pain or burning sensation when you are urinating.
D5	Pain or burning sensation within the 30 minutes after urinating.
D6	Pain or discomfort around the urethra when you are not urinating.
D7	Pain or discomfort in your pelvis or lower tummy/abdomen (including bladder pressure).
D8	Pain or discomfort in your lower back.
D9	Pain or discomfort in your side/flank.
D10	Pain or discomfort radiating down into your legs.
Section E: Critical clinical features	Please indicate whether you:
E1	Have diabetes (of any type).
E2	Have used any type of catheterisation to drain your bladder in the past week.
E3	Have experienced constipation in the past 24 hours.
Instruction	The following questions are specific to females and people assigned female at birth. If applicable, please indicate whether you are currently:
E4	Pregnant.
E5	Experiencing vaginal bleeding (e.g., period/menstruation, spotting, perimenopausal bleeding).
E6	Experiencing premenstrual symptoms (e.g., tummy pain or cramps).
E7	Experiencing menopausal or perimenopausal symptoms (e.g., vaginal dryness or pain, hot flushes, night sweats).

*Note*: A typeset version of the Recurrent Urinary Tract Infection Symptom Scale is available in Figure S5. Scale response option information: Section A uses a yes/no option for item A1, and items A2 and A3 require a numeric response (only required for respondents who select ‘no’ for item A1); Section B uses an 11‐point global rating of change scale ranging from −5 (*very much worse*) to 0 (*no change*) to +5 (*very much better*); Sections C and D use an 11‐point scale ranging from 0 (*not present*) to 1 (*very mild*) to 10 (*extremely severe*); Section E uses yes/no options for all items.

#### 3.3.3 Internal consistency

Cronbach's alpha (α) ranged from good (0.80–0.90) to excellent (0.90–1.00) for both the 7‐item *urinary symptoms* and 10‐item *UTI pain* subscales (α = 0.87 and α = 0.93, respectively), meeting the COSMIN minimum recommendation of α = 0.70.^18^ The RUTISS as a whole scale achieved excellent internal consistency (α = 0.94).^18^

#### 3.3.4 Test–retest reliability

Both the *urinary symptoms* and *UTI pain* subscales were statistically significantly stable between the Baseline and Test–Retest Assessments, with intraclass correlation coefficients (ICC) greater than the minimum recommendation 0.70 (*urinary symptoms*: ICC = 0.73, 95% CI [0.59, 0.82], *p* < 0.001; *UTI pain*: ICC = 0.82, 95% CI [0.75, 0.88], *p* < 0.001).^18^

ICC were also calculated for individual items, with a mean of 0.68. All items achieved at least a moderate ICC (ICC > 0.50) which was statistically significant at *p* < 0.001, apart from the GRC scale (ICC = 0.33, *p* < 0.001) and the item assessing current pain intensity (ICC = 0.07, *p* = 0.20).^18^ Given rapid fluctuation and variability in UTI symptoms over time, described by both expert clinicians and patients who participated during the development stages, it is reasonable that these two items may be different over this test–retest window, thus the results for these two items are clinically normative. Further, this supports the rationale for the questionnaire's 24‐h recall period, because any longer may cover excessive symptom variation.

#### 3.3.5 Construct validity

The *urinary symptoms* subscale correlated most strongly with the UTISA scores, demonstrating a statistically significant strong correlation with acute UTI symptoms (UTISA: Spearman's ρ = 0.77, *p* < 0.01; NPRS: Spearman's ρ = 0.60, *p* < 0.01).^18^ The *UTI pain* subscale correlated most strongly with the NPRS scores, also achieving a statistically significant strong correlation (UTISA: Spearman's ρ = 0.75, *p* < 0.01; NPRS: Spearman's ρ = 0.82, *p* < 0.01). Therefore, both these subscales efficiently measure their target constructs with Spearman's ρ > 0.50, and the construct validity hypotheses were satisfied.^18^

#### 3.3.6 Global rating of change scale

Linear regression analysis confirmed that the GRC scale was found to statistically significantly negatively predict both RUTISS subscales. The GRC scale explained 12.4% of the variance in *urinary symptom* subscale scores (*R* ^2^_Adj_ = 0.12, *F*(1, 238) = 34.8, *p* < 0.001) and negatively predicted symptom severity (β = −2.45, *p* < 0.001, 95% CI [−3.27, −1.63]). The GRC scale explained 13.8% of the variance in *UTI pain* subscale scores (*R* ^2^_Adj_ = 0.14, *F*(1, 238) = 39.1, *p* < 0.001) and negatively predicted pain severity (β = −3.50, *p* < 0.001, 95% CI [−4.61, −2.40]). Overall, lower GRC scores (demonstrating a worsening of symptoms) capably predicted greater symptom and pain severity scores.

#### 3.3.7 Demographic variability

There were no statistically significant group differences in RUTISS scores in terms of gender, ethnicity, country of residence, or relationship status (*p* > 0.05), indicating the broad sociocultural applicability of the RUTISS. Linear regression analysis indicated that age statistically significantly negatively predicted both RUTISS subscale scores, suggesting that younger adults typically reported higher symptom severity than older adults in this sample, explaining 3.10% of the variance in *urinary symptom* severity (*R* ^2^_Adj_ = 0.03, *F*(1, 238) = 8.76, *p* < 0.01) and 3.60% of the variance in *UTI pain* severity (*R* ^2^_Adj_ = 0.04, *F*(1, 238) = 9.97, *p* < 0.01).

#### 3.3.8 Readability

The Automated Readability Index for the RUTISS is 7.0, indicating that this measure is suitable for people aged 12 years old and above (7th US grade, equivalent to UK Key Stage 3/year 8).^30^

## 4 DISCUSSION

This study developed and validated the first PROM of rUTI symptom severity, the Recurrent UTI Symptom Scale, providing clinicians, researchers, and healthcare organisations with the first ever validated, clinically informed and patient‐evaluated measure of patient‐reported symptoms and pain. The final 28‐item questionnaire includes an assessment of UTI symptom frequency, a global rating of change scale, a *urinary symptoms* subscale, a *UTI pain* subscale, and an additional section evaluating critical clinical features such as diabetes and pregnancy. The identified four‐factor structure capably distinguishes between urinary symptoms and UTI pain, and demonstrates excellent reliability and validity, meeting or surpassing all applicable COSMIN recommendations.^17^, ^18^ It is recognised that symptoms are not the only indicators of rUTI burden, therefore it is recommended that the RUTISS be administered alongside condition‐specific measures of quality of life such as the Recurrent UTI Impact Questionnaire.^31^

The development of the RUTISS included in‐depth input from heterogeneous international expert clinician and patient samples, employing a mixed‐methods design which rigorously followed COSMIN's PROM development guidance and allowed for iterative refinement.^17^, ^18^ The RUTISS was validated using a large international sample of patients, also following COSMIN recommendations.^17^, ^18^ The observed reliability and validity statistics demonstrate the strong psychometric properties and readability of the RUTISS, indicating its promise for effective application to both clinical and research settings. Further, the broad demographic and clinical diversity of both patient samples supports the generalisability of the results across the range of rUTI patient experiences representative of this underexplored patient cohort.

Although rUTI is considerably more prevalent in females than in males,^4^ further testing would be beneficial to specifically assess the reliability and validity of the RUTISS in males or people identifying as non‐binary. It is also acknowledged that most patient participants in this study were Caucasian, native English speakers residing in high‐income countries. Further research is necessary to establish cross‐ validation of this new measure, including evaluating its sensitivity to specific rUTI patient sub‐cohorts (such as people with diabetes, immunosuppression, neurogenic urinary tract dysfunction, and those using urinary catheterisation). Future research could also aim to compare outcomes by engaging with non‐recurrent UTI populations and other urological presentations (such as bladder pain syndrome). Additional research to examine the feasibility of developing a short‐form version of the RUTISS may be beneficial.

The RUTISS is the first PROM to specifically assess the rUTI patient experience and prioritise the importance of rUTI self‐report, going beyond the UTISA and ACSS which are validated to evaluate acute UTI and acute cystitis, respectively.^15^, ^16^ The RUTISS additionally captures symptoms more typically associated with a recurrent experience of UTI, further examining symptom frequency and a global rating of change scale. The use of the familiar 11‐point scale in the RUTISS is also superior to the otherwise used 4‐point scale, known to perform relatively poorly on measures of reliability, validity and discriminating power.^19^ There is limited data on the psychometric properties of the UTISA and ACSS, however the RUTISS attained strong quantitative indicators of measure strength. The RUTISS demonstrated stronger internal consistency and construct validity than related acute UTI and cystitis measures.

Reliable, valid measures of rUTI are essential to improving our understanding and treatment of this debilitating condition, ultimately improving patient outcomes. The lack of capacity to measure the patient experience of rUTI has been widely reported,^12^, ^13^ therefore the RUTISS represents an important step towards supplementing well‐established clinical testing methods with the patient experience. By encouraging patient‐centred care, the application of the RUTISS in clinical management allows for standardised observation, monitoring, mapping, and validation of patient‐reported outcomes.^14^ The insight gained from investigating such patient‐reported outcomes could be used to establish interventions which improve treatment provisions for people with rUTI.^12^, ^13^, ^14^ This PROM has the potential to obtain a sensitive and rapid indication of changes in symptom experience over time, and could be used to cross‐validate between clinical and medical interventions and patient outcomes.^14^ The RUTISS is a novel and strategically important measure which specifically evaluates the patient‐reported experience of rUTI, and offers a critical, data‐driven and patient‐centred assessment tool for the quantification of rUTI symptoms and pain across multiple clinical and research domains.

## DISCLOSURE OF INTEREST

Melissa Kramer is CEO of Live UTI Free; however, no financial incentives have been received.

## AUTHOR CONTRIBUTIONS

All authors contributed to the study conception and design. Material preparation and data collection for Stages I, II and III were conducted by Abigail F. Newlands. Data analysis was conducted by Abigail F. Newlands and Katherine A. Finlay. Melissa Kramer and Jessica L. Price supported participant recruitment via Live UTI Free. The original draft of the manuscript was written by Abigail F. Newlands and all authors commented on previous versions of the manuscript. All authors read and approved the final manuscript.

## Supporting information


**Figure S1.** Sampling and recruitment strategy: Cognitive interview stage

**Figure S2.** Cognitive interview topic guide

**Figure S3.** Sampling and recruitment strategy: Pilot stage

**Figure S4.** Data handling strategy: Pilot stage

**Figure S5.** Recurrent UTI Symptom Scale (RUTISS)

**Table S1.** Content validity indices for items, qualitative feedback, and refinements: Expert screening stage

**Table S2.** Qualitative feedback and refinements: Cognitive interview stage
