# Supplementary material for: The Recurrent Urinary Tract Infection Symptom Scale: Development and validation of a patient‐reported outcome measure
Source: BJUI Compass. 2023 Jan 17;4(3):285–97. doi: 10.1002/bco2.222 (PMC10071086; doi:10.1002/bco2.222)
Supplement: Supplementary file 1 — Figure S1. Sampling and recruitment strategy: Cognitive interview stage [file BCO2-4-285-s005.pdf]

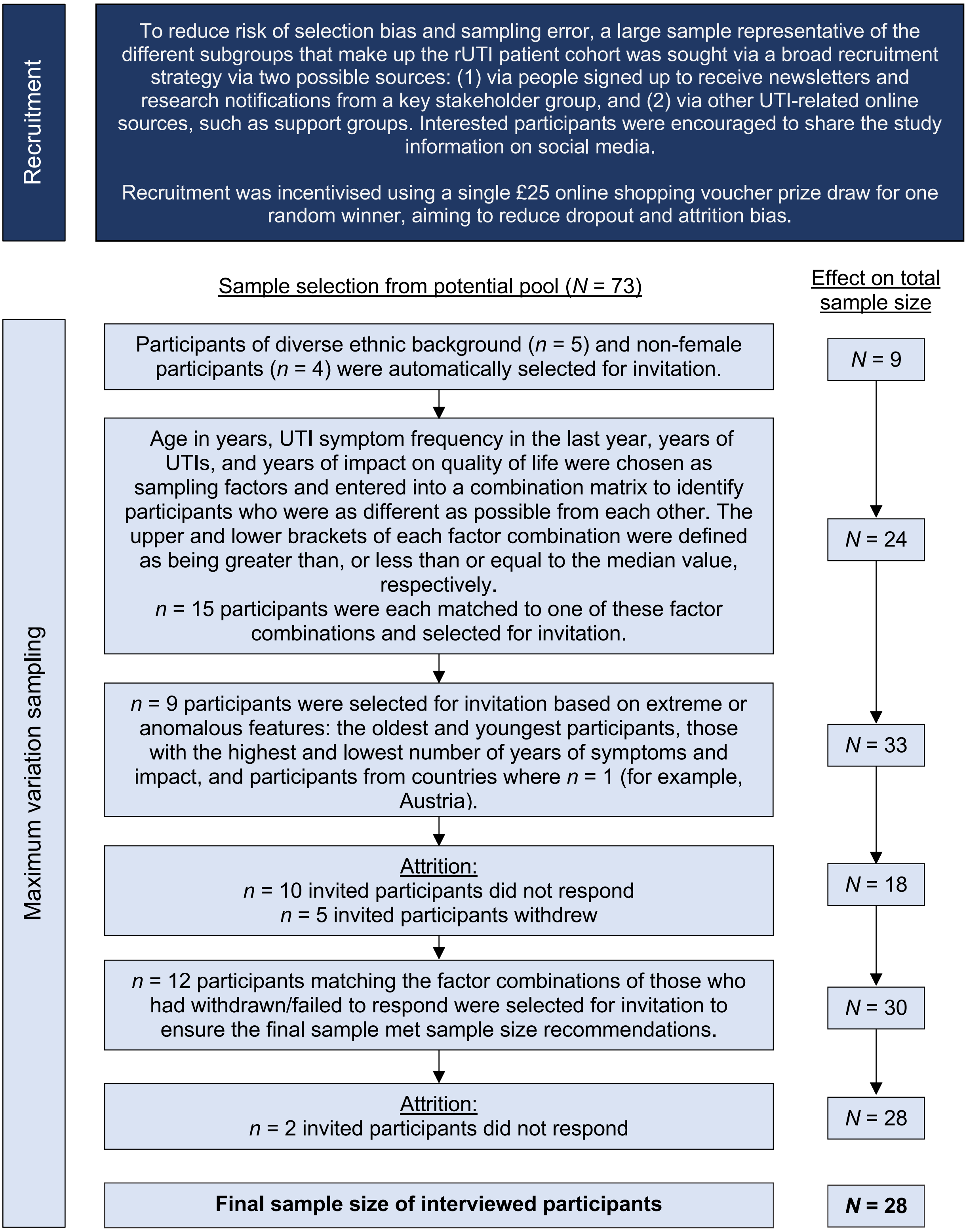

Note. A total of 73 potential participants completed the consent form and screening survey without being excluded. A maximum variation sampling strategy was applied to select as diverse a sample as possible from this potential pool, as described above.(25) This resulted in a final sample of 28 interviewed patient participants.
