# Supplementary material for: The Recurrent Urinary Tract Infection Symptom Scale: Development and validation of a patient‐reported outcome measure
Source: BJUI Compass. 2023 Jan 17;4(3):285–97. doi: 10.1002/bco2.222 (PMC10071086; doi:10.1002/bco2.222)
Supplement: Supplementary file 2 — Figure S2. Cognitive interview topic guide [file BCO2-4-285-s003.pdf]

Topic Guide for Cognitive Interviews: Development of the Recurrent UTI Symptom Scale (RUTISS)

Use "think aloud" and "verbal probing" techniques following Willis' guidelines.[1]

1. **INTRO:**
   - What are your initial impressions of the questionnaire?
   - UTI definition:
     - How easy or difficult is this to understand?
     - How useful is it to have this definition?

2. **SECTION A: FREQUENCY OF UTI SYMPTOMS**

General probing questions:

- How easy or difficult is this to answer?
- What do you think the question is trying to find out?
- I noticed that you hesitated – what were you thinking about?
- How did you feel about the requirement to think about your symptoms over the past 24 hours? / What do you think the best timeline would be for assessing symptoms?
- How do you feel about the format of the questions?
- Is there anything you would change to make it better?

Possible question/section-specific probing questions:

- **A1:** What does "one non-stop UTI with continuous symptoms" mean to you?
- How easy is this instruction to follow? ("If you selected "yes", please skip…")
- **A2** and **A3**: How do you feel about the inclusion of the definition of "episodes" here?
- **A2** and **A3**: How did you work out your answer of X episodes in 6/12 months?
- Do you feel that these questions are appropriate for determining the frequency of your UTI symptoms?

3. **SECTION B: URINARY SYMPTOMS**

General probing questions:

- How easy or difficult is this to answer?
- What do you think the question is trying to find out?
- I noticed that you hesitated – what were you thinking about?
- How did you feel about the requirement to think about your symptoms over the past 24 hours? / What do you think the best timeline would be for assessing symptoms?
- How do you feel about the format of the questions?
- Is there anything you would change to make it better?

Possible question/section-specific probing questions:

- How do you interpret "very mild" symptoms versus "worst imaginable" severity?
- How familiar are you with the different symptoms listed here?
- Is there any symptom on this list that you feel shouldn't be here?
- Is there anything you feel is missing from this list of symptoms?

4. **SECTION C: UTI-RELATED PAIN**

General probing questions:

- How easy or difficult is this to answer?
- What do you think the question is trying to find out?
- I noticed that you hesitated – what were you thinking about?
- How did you feel about the requirement to think about your symptoms over the past 24 hours? / What do you think the best timeline would be for assessing symptoms?
- How do you feel about the format of the questions?
- Is there anything you would change to make it better?

Possible question/section-specific probing questions:

- What does "very mild" pain versus "worst imaginable pain" mean to you?
- **C1** and **C2**: What do you think is the difference between C1 and C2? / Can you repeat the question in your own words?
- **C1** and **C2**: How did you work out your average level of pain?
- **C5**: What does "after urinating" mean to you?
- **C6**: What does "around the urethra" mean to you?
- **C7**: What does "pelvis or lower tummy/abdomen" mean to you?
- **C8**: What does "side/flank" mean to you?
- Is there any aspect of pain on this list that you feel shouldn't be here?
- Is there anything you feel is missing from this list of types of UTI-related pain?

5. **SECTION D: FINAL QUESTIONS**

General probing questions:

- How easy or difficult is this to answer?
- What do you think the question is trying to find out?
- I noticed that you hesitated – what were you thinking about?
- How did you feel about the requirement to think about your symptoms over the past 24 hours? / What do you think the best timeline would be for assessing symptoms?
- How do you feel about the format of the questions?
- Is there anything you would change to make it better?

Possible question/section-specific probing questions:

- How do you feel about answering these questions?
- How do you interpret "not applicable" here?

6. **OVERALL QUESTIONNAIRE**
   - What are your overall views on the questionnaire?
   - Is there anything you would change to make it better?
   - Is there anything else you'd like to say about this questionnaire that I haven't already asked?
   - How do you feel about the amount of time that these questions take to answer?

[1] Willis G. Cognitive Interviewing: A "How To" Guide. Rockville: Research Triangle Institute; 1999.
