# Supplementary material for: The Recurrent Urinary Tract Infection Symptom Scale: Development and validation of a patient‐reported outcome measure
Source: BJUI Compass. 2023 Jan 17;4(3):285–97. doi: 10.1002/bco2.222 (PMC10071086; doi:10.1002/bco2.222)
Supplement: Supplementary file 3 — Figure S3. Sampling and recruitment strategy: Pilot stage [file BCO2-4-285-s006.pdf]

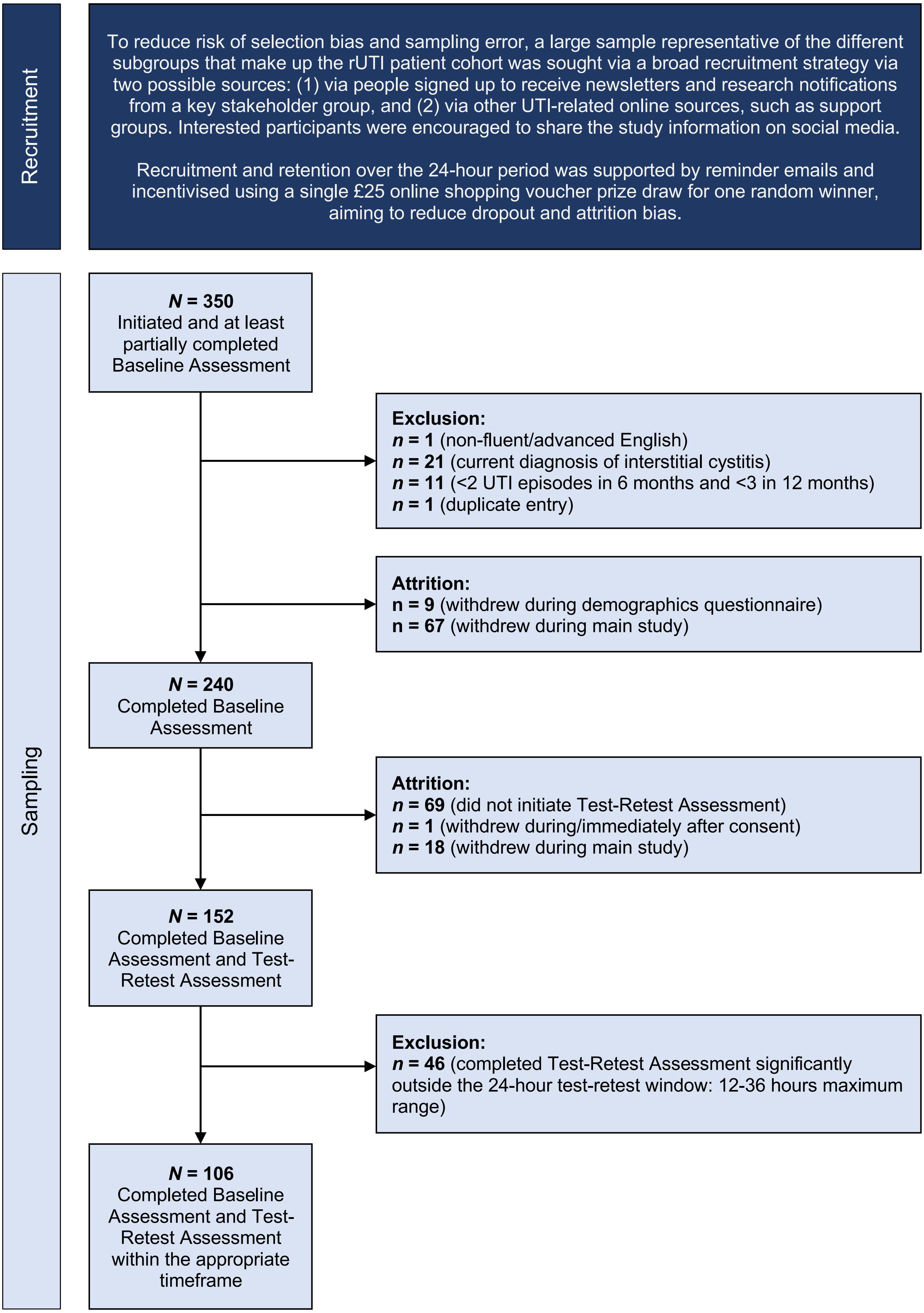

Note. Data from all 240 participants who completed the Baseline Assessment were included in every statistical test, except for the test-retest analyses which were conducted only with those who completed both the Baseline and Test-Retest Assessments (*N* = 106). Participants with missing data (for example, those who withdrew from the Baseline and/or Test-Retest Assessment without completion) were excluded from analysis.
