# Supplementary material for: The Recurrent Urinary Tract Infection Symptom Scale: Development and validation of a patient‐reported outcome measure
Source: BJUI Compass. 2023 Jan 17;4(3):285–97. doi: 10.1002/bco2.222 (PMC10071086; doi:10.1002/bco2.222)
Supplement: Supplementary file 4 — Figure S4. Data handling strategy: Pilot stage [file BCO2-4-285-s004.pdf]

Data preparation and assumption testing

1. Total scores were calculated for each of the Recurrent Urinary Tract Infection Symptom Score (RUTISS) subscales at Baseline and Test-Retest Assessments. Total scores for the RUTISS as a whole measure were not computed to avoid misrepresenting multidimensional constructs as a single score.[1]

2. Data collected using the Urinary Tract Infection Symptom Assessment (UTISA) and Numerical Pain Rating Scale (NPRS) were scored according to their standard scoring systems.

3. The following assumptions were assessed:
   - Normality (Shapiro-Wilk $p>.05$ so non-parametric analyses were conducted where applicable)
   - Linearity (examination of scatter plots confirmed no violation)
   - Multicollinearity (Variance Inflation Factor scores for the target label data as well as possible demographic characteristics were sufficiently close to 1)
   - Independence of observations (Durbin-Watson statistic was approximately 2 for each subscale score)
   - Homoscedasticity (inspection of the standardised residuals plotted against standardised predicted values confirmed no violation)

4. Case-wise diagnostics of the subscale scores indicated five possible outliers outside 3SD; however, since these were not measurement errors and could feasibly reflect true variation within the population, they were not removed. Analyses conducted with and without these cases indicated that they had no statistically significant effect on the outcomes ($p > .05$).

Statistical analyses

Exploratory factor analysis (EFA):
- Suitability for EFA was checked through bivariate correlation matrices with coefficients greater than .80 examined for potential multicollinearity.[2]
- Preliminary data analyses were undertaken to assess the factorability of the dataset using Bartlett's Test of Sphericity and the Kaiser-Meyer-Olkin Measure of Sampling Adequacy.[3]
- Principal Axis Factoring was selected for application above Principal Components Analysis and Maximum Likelihood Analysis to facilitate determination of a latent factor structure.[3,4] Varimax rotation with Kaiser Normalisation was applied to minimise cross-loading of items on factors.[4]
- Visual inspection of scree plots determined the retained number of factors, combined with evaluation of eigenvalues exceeding Kaiser's criterion of 1.[5,6]
- Items with extracted communalities less than .40 were removed, and a minimum factor loading of .40 was defined as acceptable a priori.[4,5]

Internal consistency was measured for each finalised post-EFA RUTISS subscale and the RUTISS as a whole measure using Cronbach's alpha.

To assess **test-retest reliability**, intraclass correlation coefficients (ICC) and their 95% confidence intervals were computed based on a single-rating, absolute-agreement, two-way mixed effects model, as recommended for test-retest reliability analysis of measures intended to demonstrate outcomes at an individual (single-rater) level.[7]

Construct validity was assessed by computing the level of Spearman's correlation between each RUTISS subscale and the observed UTISA and NPRS scores.

Linear regression analyses were also conducted to examine whether there was a statistically significant effect of any demographic variables on the RUTISS scores (i.e., measurement invariance), and to test the predictive value of the global rating of change (GRC) scale.

An **Automated Readability Index**, known to be especially useful when applied to technical, non-narrative text,[8] was computed to estimate the literacy level required for comprehension of the RUTISS.

[1] Strauss ME, Smith GT. Construct validity: advances in theory and methodology. Annu Rev Clin Psychol. 2009;5:1-25.

[2] Field A. Discovering statistics using SPSS. 4 ed: SAGE Publications; 2013.

[3] Yong AG, Pearce S. A Beginner's Guide to Factor Analysis: Focusing on Exploratory Factor Analysis.
Tut in Quant Methods for Psychol. 2013;9(2):79-94.

[4] Fabrigar LR, Wegener DT, MacCallum RC, Strahan EJ. Evaluating the use of exploratory factor analysis in psychological research. Psychol Methods. 1999;4(3):272-99.

[5] Watkins MW. Exploratory Factor Analysis: A Guide to Best Practice. Journal of Black Psychol. 2018;44(3):219-46.

[6] Kaiser HF. The Application of Electronic Computers to Factor Analysis. Educ and Psych Measurement. 1960;20(1):141-51.

[7] Koo TK, Li MY. A Guideline of Selecting and Reporting Intraclass Correlation Coefficients for Reliability Research. Journal of Chiropractic Medicine. 2016;15(2):155-63.

[8] Kincaid J, Delionback L. Validation of the Automated Readability Index: A follow-up. Human Factors. 1973;15(1):17-20.
