# Supplementary material for: The Recurrent Urinary Tract Infection Symptom Scale: Development and validation of a patient‐reported outcome measure
Source: BJUI Compass. 2023 Jan 17;4(3):285–97. doi: 10.1002/bco2.222 (PMC10071086; doi:10.1002/bco2.222)
Supplement: Supplementary file 5 — Figure S5. Recurrent UTI Symptom Scale (RUTISS) [file BCO2-4-285-s007.pdf]

Recurrent Urinary Tract Infection Symptom Scale (RUTISS)

A urinary tract infection, or UTI, is an infection in any part of your urinary system. This may include your bladder, urethra, ureters, and/or kidneys. Some people may experience **episodes of UTI symptoms with no symptoms in between**, while some people may experience **UTI symptoms that feel continuous and do not fully subside**. This questionnaire asks about your experience of UTI symptoms and pain or discomfort.

A) The following questions are about how often you experience UTI symptoms. Please consider UTIs that may or may not have been medically diagnosed.

		Yes	No
A1.	Have you had UTI symptoms that feel continuous and do not fully subside **for at least the past 3 months**?	☐	☐

If you selected "Yes", please skip to section B. If you selected "No", please continue with the rest of Section A.

A2. Approximately how many episodes of UTI symptoms have you had in the **past 6 months**? ___________

A3. Approximately how many episodes of UTI symptoms have you had in the **past 12 months**? ___________

B) The following questions are about any change in your UTI symptoms.

B1. Please consider how you typically experience UTI symptoms. To what extent have your UTI symptoms over the PAST 24 HOURS been better or worse than your typical experience?

Very much worse					No change					Very much better
-5	-4	-3	-2	-1	0	+1	+2	+3	+4	+5
☐	☐	☐	☐	☐	☐	☐	☐	☐	☐	☐

C) The following questions are about your UTI symptoms other than pain or discomfort.

Please indicate whether you have experienced any of the following symptoms related to UTI in the PAST 24 HOURS, and if so, how SEVERE they were:

		Not present 0	Very mild 1	2	3	4	5	6	7	8	9	Extremely severe 10
C1.	Needing to urinate more frequently than normal.	☐	☐	☐	☐	☐	☐	☐	☐	☐	☐	☐
C2.	Needing to urinate more urgently or more suddenly than normal.	☐	☐	☐	☐	☐	☐	☐	☐	☐	☐	☐
C3.	Feeling as though you are unable to completely empty your bladder.	☐	☐	☐	☐	☐	☐	☐	☐	☐	☐	☐
C4.	Feeling as though you have the urge to urinate despite having just urinated.	☐	☐	☐	☐	☐	☐	☐	☐	☐	☐	☐
C5.	Urine with an unusually strong or unpleasant smell.	☐	☐	☐	☐	☐	☐	☐	☐	☐	☐	☐
C6.	Cloudy urine.	☐	☐	☐	☐	☐	☐	☐	☐	☐	☐	☐
C7.	Debris or floating particles in your urine.	☐	☐	☐	☐	☐	☐	☐	☐	☐	☐	☐

D) The following questions are about any <u>pain or discomfort in your lower abdomen, genitals and/or bladder</u>, related to your UTI(s).

		Not present	Very mild									Extremely severe
		**0**	**1**	**2**	**3**	**4**	**5**	**6**	**7**	**8**	**9**	**10**
D1.	When you are urinating, how has your pain or discomfort been **on average over the past 24 hours**?	☐	☐	☐	☐	☐	☐	☐	☐	☐	☐	☐
D2.	When you are <u>not</u> urinating, how has your pain or discomfort been **on average over the past 24 hours**?	☐	☐	☐	☐	☐	☐	☐	☐	☐	☐	☐
D3.	What is your level of pain or discomfort **right now**?	☐	☐	☐	☐	☐	☐	☐	☐	☐	☐	☐

Please indicate whether you have experienced any of the following symptoms <u>related to UTI</u> in the PAST 24 HOURS, and if so, how SEVERE they were:

		Not present	Very mild									Extremely severe
		**0**	**1**	**2**	**3**	**4**	**5**	**6**	**7**	**8**	**9**	**10**
D4.	Pain or burning sensation when you are urinating.	☐	☐	☐	☐	☐	☐	☐	☐	☐	☐	☐
D5.	Pain or burning sensation within the 30 minutes <u>after</u> urinating.	☐	☐	☐	☐	☐	☐	☐	☐	☐	☐	☐
D6.	Pain or discomfort around the urethra when you are <u>not</u> urinating.	☐	☐	☐	☐	☐	☐	☐	☐	☐	☐	☐
D7.	Pain or discomfort in your pelvis or lower tummy/abdomen (including bladder pressure).	☐	☐	☐	☐	☐	☐	☐	☐	☐	☐	☐
D8.	Pain or discomfort in your lower back.	☐	☐	☐	☐	☐	☐	☐	☐	☐	☐	☐
D9.	Pain or discomfort in your side/flank.	☐	☐	☐	☐	☐	☐	☐	☐	☐	☐	☐
D10.	Pain or discomfort radiating down into your legs.	☐	☐	☐	☐	☐	☐	☐	☐	☐	☐	☐

E) Please indicate whether you:

		Yes	No
E1.	Have diabetes (of any type).	☐	☐
E2.	Have used any type of catheterisation to drain your bladder in the <u>past week</u>.	☐	☐
E3.	Have experienced constipation in the <u>past 24 hours</u>.	☐	☐

The following questions are specific to females and people assigned female at birth. If applicable, please indicate whether you are currently:

		Yes	No
E4.	Pregnant.	☐	☐
E5.	Experiencing vaginal bleeding (e.g. period/menstruation, spotting, perimenopausal bleeding).	☐	☐
E6.	Experiencing premenstrual symptoms (e.g. tummy pain or cramps).	☐	☐
E7.	Experiencing menopausal or perimenopausal symptoms (e.g. vaginal dryness or pain, hot flushes, night sweats).	☐	☐

Thank you. This is the end of the questionnaire.
