# Supplementary material for: The Recurrent Urinary Tract Infection Symptom Scale: Development and validation of a patient‐reported outcome measure
Source: BJUI Compass. 2023 Jan 17;4(3):285–97. doi: 10.1002/bco2.222 (PMC10071086; doi:10.1002/bco2.222)
Supplement: Supplementary file 6 — Table S1. Content validity indices for items, qualitative feedback, and refinements: Expert screening stage [file BCO2-4-285-s001.docx]

### **Supplementary Table 1**

### Content validity indices for items, qualitative feedback, and refinements made during expert screening study

| Original instruction/item | Updated instruction/item | Quotation(s) | Round 1 | | | | Round 2 | | | |
| --- | --- | --- | --- | --- | --- | --- | --- | --- | --- | --- |
|  |  |  | Relevance | | Clarity | | Relevance | | Clarity | |
|  |  |  | *Mdn* | I-CVI | *Mdn* | I-CVI | *Mdn* | I-CVI | *Mdn* | I-CVI |
| Section A |  |  |  |  |  |  |  |  |  |  |
| The following questions relate to the frequency of your urinary tract infections (UTIs). | The following questions are about how often you experience UTIs. Please consider UTIs that may or may not have been medically diagnosed. | GP (UK): “some patients may not understand 'frequency' so may be worth adding in brackets 'how often they happen' - and again not everyone is familiar with urinary tract infections - is it with clarifying with 'urine infections / cystitis' to put it in lay terms?”  Urogynaecologist (USA): “Syntax could be a bit complex for some readers. Would consider: The following questions relate to how often your urinary tract infections happen. Or... how often your UTIs occur. Or... how often you experience urinary tract infections”  Urogynaecologist (UK): “Needs to be clear [that] this means diagnosed or not diagnosed.” | − | − | 6 | 1.00 | − | − | 6 | 1.00 |
| A1. Approximately how many UTIs do you feel you have had in the last 6 months? | Approximately how many episodes of UTI symptoms have you had in the past 6 months? | GP (USA): “Clarify times they have had symptoms vs. culture-proven infections i.e., how many times do you feel you have had symptoms of UTI?”  Urogynaecologist (USA): “Would change the syntax "do you feel you have had" to something closer to 8th grade level”  Urology nurse practitioner (USA): “This going on the patient perspective of how many UTIs they think they have had based on their symptoms so I think this is appropriate” | 6 | 1.00 | 6 | .93 | 6 | 1.00 | 6 | 1.00 |
| A2. Approximately how many UTIs do you feel you have had in the last 12 months? | Approximately how many episodes of UTI symptoms have you had in the past 12 months? | [as above in A1] | 6 | 1.00 | 6 | 1.00 | 6 | 1.00 | 6 | 1.00 |
| NEW ITEM | [to go before A1 and A2]  Do you feel you have had one non-stop UTI with continuous symptoms for the past 3 months or more? (Yes/No) | Urologist (USA): “Patients who think they have a UTI ‘all the time’ won’t be able to respond.”  GP (UK): “I think patients need the option of choosing A3 Do you feel you have had one unremitting UTI, as persistently recurrent UTI can lead to a chronic inflammatory response”  Urogynaecologist (USA): “I think it is reasonable to consider whether patients perceive they have chronic symptoms and to assess this differently.”  Urologist (USA): “I agree with the … comment about "unremitting UTI".  GP (UK): “For patients who have persistently recurrent UTI they may have had what they perceive to be one long chronic UTI lasting throughout the previous 6 months.” | − | − | − | − | − | − | − | − |
| NEW INSTRUCTION | [to go after new item, before A1 and A2]  If you selected “Yes”, please skip to section B. If you selected “No”, please continue with the rest of Section A. | [as above] | − | − | − | − | − | − | − | − |
| NEW INSTRUCTION/EXPLANATION | [to go before A1 and A2]  The term “episode” is used here to describe a temporary increase in UTI symptoms before they return to a level that is normal for you. | Urogynaecologist (USA): “Some people with more chronic symptoms may not know how to answer this without a better definition of what is meant by UTI.” | − | − | − | − | − | − | − | − |

*Note.* *Mdn* = Median rating. I-CVI = item content validity index.

Round 1 *n* = 15. Round 2 *n* = 12.

Instructions were tested only for clarity of wording. Items were tested for both clarity and relevance for recurrent UTI.

Median ratings < 4 are in bold. I-CVI < .75 are in bold. I-CVI = 1.00 indicates that all expert participants rated the item/instruction as at least 4 out of 6 (where 6 = highly relevant/clear).

Updated items were taken forward for testing in the first phase of patient cognitive interviews. Hyphens (−) indicate that no relevance or clarity ratings were obtained (for example, for new items or instructions added after expert screening, or for instructions which were only tested for clarity), or that no changes were made.

| Original instruction/item | Updated instruction/item | Quotation(s) | Round 1 | | | | Round 2 | | | |
| --- | --- | --- | --- | --- | --- | --- | --- | --- | --- | --- |
|  |  |  | Relevance | | Clarity | | Relevance | | Clarity | |
|  |  |  | *Mdn* | I-CVI | *Mdn* | I-CVI | *Mdn* | I-CVI | *Mdn* | I-CVI |
| Section B |  |  |  |  |  |  |  |  |  |  |
| The following questions relate to your non-pain related UTI symptoms. | The following questions are about your UTI symptoms other than pain. | GP (USA): “I am unclear what the non-pain related symptoms are and saying non pain related confuses me”  Urologist (USA): “There are numerous non-pain symptoms that might not actually be related to a rUTI so the question should be specific” | − | − | 6 | .86 | − | − | 6 | .83 |
| Please indicate whether you had any of the following non-pain related symptoms in the past 24 hours and if so, how SEVERE they were: | Please indicate whether you had any of the following symptoms in the PAST 24 HOURS, and if so, how SEVERE they were: | [avoid repetition] | − | − | 6 | .86 | − | − | 6 | .83 |
| B1. Needing to urinate more urgently or more suddenly than normal. | − | − | 6 | 1.00 | 6 | .87 | 6 | 1.00 | 6 | .92 |
| B2. Needing to urinate more frequently than normal. | − | − | 6 | 1.00 | 6 | 1.00 | 6 | 1.00 | 6 | 1.00 |
| B3. Unintentionally passing urine (e.g. due to urgency). | Unintentionally passing or leaking urine. | Urogynaecologist (USA): “Are you referring to incontinence here? Unintentionally leaking instead of unintentionally passing?”  Urogynaecologist (USA): “passing or leaking”  Urologist (USA): “I would say leakage instead of "passing urine" at least in the USA”  GP (Canada): “I agree with changing it to leakage” | 6 | 1.00 | 5 | **.67** | 6 | 1.00 | 4 | .75 |
| B4. Feeling as though you are unable to empty your bladder fully. | Feeling as though you are unable to completely empty your bladder. | Urogynaecologist (USA): “? Completely empty your bladder?”  Urologist (USA): “Prefer "feeling of incomplete emptying" after peeing” | 6 | .80 | 6 | 1.00 | 6 | .83 | 6 | 1.00 |
| B5. Blood in your urine. | Visible blood in your urine. | Urologist (USA): “I would ask about blood you can see in the urine since many women will report microscopic hematuria that was seen on dipstick or UA from the MD”  Urogynaecologist (UK): “Patients often use dipsticks themselves. I would ask if they have 'seen' blood in the urine.”  Urogynaecologist (USA): “I would specify SEEING blood, cause often patients have some awareness of having been told by a doctor that they had blood on a dipstick or in a microscopic urinalysis.” | 6 | .93 | 6 | .80 | 6 | .92 | 6 | 1.00 |
| B6. Cloudy urine. | - | - | 5 | **.67** | 6 | .87 | **3** | **.42** | 5.5 | **.67** |
| B7. Change in your temperature (fever or chills). | [Split into two questions]  1. Fever (feeling hot with a temperature higher than 38°C or 100.4°F).  2. Chills (feeling cold and shivery). | Urogynaecologist (USA): “Change in temperature again seems needlessly complicated. I would separate fever and chills. Define fever with a specific cut off (so you don't have people saying, well I am normally 35 degrees, so 35.5 degrees is a fever for me). Chills are subjective, fever is not, I would not combine them.” | 5 | **.67** | 6 | .87 | 4.5 | **.67** | 6 | .92 |
| B8. Constipation. | [Moved to Section D as confound] | Urogynaecologist (USA): “Constipation has a lot of meanings. It is not a feature of UTI, but a feeling of irritation and needing to strain to defecate is.”  GP (Canada): Not very specific to an acute UTI episode in terms of symptomology. Definitely a potentially contributing cause though … Could consider adding constipation [to Section D] instead of in section B.”  Urology nurse practitioner (USA): “Constipation is a risk factor for recurrent UTI and lower urinary tract symptoms. It can lead to incomplete bladder emptying and in turn cause bacteriuria. It is relevant.” | 5 | **.67** | 5 | **.67** | 4 | **.58** | 5 | **.67** |
| Scale: 0 = not present; 1 = very mild; 10 = worst imaginable | − | − | − | − | − | − | − | − | − | − |
| NEW ITEM | Feeling as though you have the urge to urinate despite having just urinated. | Urology nurse practitioner (USA): “along the same lines as the urgency, feeling as though you have to urinate even after you just did” | − | − | − | − | − | − | − | − |
| NEW ITEM | Urine with an unusually strong or unpleasant smell. | GP (UK): “is there a place for smelly /offensive urine or is that not considered sufficiently diagnostic? I would think it adds to the evidence of possible UTI”  GP (Canada): “Urine odour … could be considered in this section”  Urologist (USA): “I would agree that smelly urine is indicative” | − | − | − | − | − | − | − | − |
| NEW ITEM | Debris or floating particles in your urine. | GP (Canada): “Debris in urine could be considered in this section” | − | − | − | − | − | − | − | − |

*Note.* *Mdn* = Median rating. I-CVI = item content validity index.

Round 1 *n* = 15. Round 2 *n* = 12.

Instructions were tested only for clarity of wording. Items were tested for both clarity and relevance for recurrent UTI.

Median ratings < 4 are in bold. I-CVI < .75 are in bold. I-CVI = 1.00 indicates that all expert participants rated the item/instruction as at least 4 out of 6 (where 6 = highly relevant/clear).

Updated items were taken forward for testing in the first phase of patient cognitive interviews. Hyphens (−) indicate that no relevance or clarity ratings were obtained (for example, for new items or instructions added after expert screening, or for instructions which were only tested for clarity), or that no changes were made.

| Original instruction/item | Updated instruction/item | Quotation(s) | Round 1 | | | | Round 2 | | | |
| --- | --- | --- | --- | --- | --- | --- | --- | --- | --- | --- |
|  |  |  | Relevance | | Clarity | | Relevance | | Clarity | |
|  |  |  | *Mdn* | I-CVI | *Mdn* | I-CVI | *Mdn* | I-CVI | *Mdn* | I-CVI |
| Section C |  |  |  |  |  |  |  |  |  |  |
| The following questions relate to your experience of UTI-related pain. | The following questions are about any pain or discomfort related to your UTI(s). | GP (UK): “I would say pain related to UTI rather than UTI-related pain - but maybe I'm just being pedantic!”  Urogynaecologist (USA): “Other thing to wonder about is pain is very differently defined for different people. You might think about including "or discomfort" as many people are uncomfortable, but don't necessary use the word "PAIN" to describe that sensation.” | − | − | 6 | 1.00 | − | − | 6 | 1.00 |
| C1. With urination, what has been your average level of pain over the past 24 hours? | When you are urinating, how has your lower abdominal, genital or bladder pain been on average over the past 24 hours? | GP (UK): “passing urine, rather than urination”  GP (UK): “I would put… ‘when you pass urine’ rather than with urination”  Urogynaecologist (USA): “Disagree with qualitative feedback about "when you pass urine" as this phrasing is more common in the UK and will not be easily interpreted in the US”  GP (UK): “Excellent choice of question, might suggest to define further by saying, lower abdominal, genital or bladder pain. Many of these patients have co-morbidities such as fibromyalgia to which they may refer to.”  Urogynaecologist (USA): “Do you mean only pain in [the] pelvis? Or pain in any area of the body? Would be more specific here.” | 6 | 1.00 | 6 | .93 | 6 | 1.00 | 6 | 1.00 |
| C2. When you are not urinating, what has been your average level of pain over the past 24 hours? | When you are not urinating, how has your lower abdominal, genital or bladder pain been on average over the past 24 hours? | [as above for C1] | 6 | 1.00 | 6 | **.73** | 6 | 1.00 | 5 | .83 |
| C3. What is your current level of pain? | What is your level of lower abdominal, genital or bladder pain right now? | [as above for C1]  Urologist (USA): “The pain is not specific to suprapubic region or dysuria. many women with myofascial issues that are not UTI have pain. This should be limited to a pain symptom associated with UTI”  GP (UK): “right now” | 6 | 1.00 | 6 | **.73** | 6 | 1.00 | 5 | **.75** |
| Scale: 0 = not present; 1 = very mild; 10 = worst imaginable pain | − | − | − | − | − | − | − | − | − | − |
| Please indicate whether you have experienced any of the following symptoms in the past 24 hours, and if so, how SEVERE they were: | Please indicate whether you have experienced any of the following symptoms related to UTI in the PAST 24 HOURS, and if so, how SEVERE they were: | Urologist (USA): “The pain is not specific to suprapubic region or dysuria. many women with myofascial issues that are not UTI have pain. This should be limited to a pain symptom associated with UTI” | − | − | 6 | 1.00 | − | − | 6 | 1.00 |
| C4. Pain or burning sensation during urination. | Pain or burning sensation when you are urinating. | GP (UK): “when passing urine, not during urination” | 6 | 1.00 | 6 | 1.00 | 6 | 1.00 | 6 | 1.00 |
| C5. Pain or burning sensation after urination. | Pain or burning sensation after urinating. | Urogynaecologist (USA): “May want to highlight or underline "after" to emphasize the wording change compared to prior question”  GP (Canada): “possibly highlight after” | 6 | 1.00 | 6 | 1.00 | 6 | 1.00 | 6 | 1.00 |
| C6. Urethral pain unrelated to urination. | Pain or discomfort around the urethra when you are not urinating. | GP (UK): “if rewording, it could be re-expressed as 'urethral pain at times when you are not passing urine'” | 6 | 1.00 | 5 | .87 | 6 | 1.00 | 5 | .83 |
| C7. Pain in your pelvis or lower tummy/abdomen. | Pain or discomfort in your pelvis or lower tummy/abdomen. | Urogynaecologist (USA): “Other thing to wonder about is pain is very differently defined for different people. You might think about including "or discomfort" as many people are uncomfortable, but don't necessary use the word "PAIN" to describe that sensation.” | 6 | 1.00 | 6 | 1.00 | 6 | 1.00 | 6 | 1.00 |
| C8. Pain in your side/flank. | Pain or discomfort in your side/flank. | [as above for C7] | 6 | .93 | 6 | 1.00 | 6 | .92 | 6 | 1.00 |
| C9. Pain in your back. | Pain or discomfort in your lower back. | [as above for C7]  GP (UK): “Patients perceive their back (correctly) to be any part of their back from shoulders to hips, may lead to confusion. Lower back more relevant / clear with respect to recurrent UTI”  Urogynaecologist (USA): “Back is really vague. Is there a specific type of back pain you are trying to get at? Paired with the flank pain question, people might not understand what kind of pain this is getting at or they will answer yes to both, so I would try to be more specific.” | 6 | .93 | 6 | .80 | 5.5 | .92 | 5.5 | .75 |
| Scale: 0 = not present; 1 = very mild; 10 = worst imaginable | − | − | − | − | − | − | − | − | − | − |

*Note.* *Mdn* = Median rating. I-CVI = item content validity index.

Round 1 *n* = 15. Round 2 *n* = 12.

Instructions were tested only for clarity of wording. Items were tested for both clarity and relevance for recurrent UTI.

Median ratings < 4 are in bold. I-CVI < .75 are in bold. I-CVI = 1.00 indicates that all expert participants rated the item/instruction as at least 4 out of 6 (where 6 = highly relevant/clear).

Updated items were taken forward for testing in the first phase of patient cognitive interviews. Hyphens (−) indicate that no relevance or clarity ratings were obtained (for example, for new items or instructions added after expert screening, or for instructions which were only tested for clarity), or that no changes were made.

| Original instruction/item | Updated instruction/item | Quotation(s) | Round 1 | | | | Round 2 | | | |
| --- | --- | --- | --- | --- | --- | --- | --- | --- | --- | --- |
|  |  |  | Relevance | | Clarity | | Relevance | | Clarity | |
|  |  |  | *Mdn* | I-CVI | *Mdn* | I-CVI | *Mdn* | I-CVI | *Mdn* | I-CVI |
| Section D |  |  |  |  |  |  |  |  |  |  |
| Finally, please indicate whether you: | − | − | − | − | 6 | .93 | − | − | 6 | .92 |
| D1. Are on your period (menstruating). | Are on your period/menstruating or experiencing other vaginal bleeding (e.g. spotting, perimenopausal bleeding). | GP (UK): “This will not take into account women who are perimenopausal or may have endometriosis (this cohort is very prone to UTI) and getting occasional dysfunctional uterine bleeding which is not actually a period but patients see blood and become confused in how to describe their symptom” | 6 | 1.00 | 6 | .93 | 6 | 1.00 | 6 | 1.00 |
| D2. Are experiencing premenstrual symptoms. | Are experiencing premenstrual symptoms (e.g. tummy pain or cramps). | Urogynaecologist (UK): “What are those symptoms? Not clear.”  Urogynaecologist (USA): “I might simplify the language here. I don't know how universally well understood the idea of "PMS" is for people.”  GP (USA): “May want to list examples of PMS symptoms” | 6 | .93 | 5 | .80 | 6 | .92 | 5 | .75 |
| D3. Are experiencing menopausal symptoms. | Are experiencing menopausal symptoms (e.g. vaginal dryness or pain,  hot flushes, night sweats). | Urogynaecologist (UK): “Again, what does this mean? menopausal symptoms are very broad. I would ask about vaginal dryness etc.”  Urogynaecologist (USA): “So many preconceived ideas about what these might be. Most people don't know urethral pain is a sign of menopause and only think of it as hot flashes and mood swings”  GP (USA): “Same comment as previous question (give examples of symptoms)” | 6 | .93 | 5 | **.67** | 5.5 | .92 | 4.5 | **.67** |
| D4. Are pregnant. | - | - | 6 | .93 | 6 | 1.00 | 6 | 1.00 | 6 | 1.00 |
| D5. Are diabetic (any type). | Are diabetic (of any type). | [grammar] | 6 | 1.00 | 6 | .86 | 6 | 1.00 | 6 | .92 |
| D6. Have an indwelling catheter or use intermittent self-catheterisation. | Use intermittent or indwelling catheterisation to drain your bladder. | Urogynaecologist (USA): “Again I would simplify the language - "use a catheter to drain your bladder" works for both and is simpler.” | 6 | 1.00 | 6 | .93 | 6 | 1.00 | 6 | .92 |
| Responses: Yes; No; Not applicable | − | − | − | − | − | − | − | − | − | − |
| NEW INTRODUCTION | A urinary tract infection, or UTI, is an infection in any part of your urinary system. This may include your bladder, urethra, ureters, and/or kidneys. | Urogynaecologist (USA): “Is there any preface to define UTIs? I have patients who think that there is a difference between UTI and bladder infection and as much as it seems like that should be evident, there may be a need to place some kind of definition in the structure.” | − | − | − | − | − | − | − | − |
| NEW ITEM | [moved from Section B]  Have experienced constipation in the past 24 hours. | GP (Canada): “Could consider adding constipation here instead of in section B.” | − | − | − | − | − | − | − | − |

*Note.* *Mdn* = Median rating. I-CVI = item content validity index.

Round 1 *n* = 15. Round 2 *n* = 12.

Instructions were tested only for clarity of wording. Items were tested for both clarity and relevance for recurrent UTI.

Median ratings < 4 are in bold. I-CVI < .75 are in bold. I-CVI = 1.00 indicates that all expert participants rated the item/instruction as at least 4 out of 6 (where 6 = highly relevant/clear).

Updated items were taken forward for testing in the first phase of patient cognitive interviews. Hyphens (−) indicate that no relevance or clarity ratings were obtained (for example, for new items or instructions added after expert screening, or for instructions which were only tested for clarity), or that no changes were made.
