# Supplementary material for: The Recurrent Urinary Tract Infection Symptom Scale: Development and validation of a patient‐reported outcome measure
Source: BJUI Compass. 2023 Jan 17;4(3):285–97. doi: 10.1002/bco2.222 (PMC10071086; doi:10.1002/bco2.222)
Supplement: Supplementary file 7 — Table S2. Qualitative feedback and refinements: Cognitive interview stage [file BCO2-4-285-s002.docx]

### **Supplementary Table 2**

Qualitative feedback and refinements made after the first phase of patient cognitive interviewing

| Instruction/item tested at Phase 1 | Updated instruction/item | Code(s) | Quotation(s) |
| --- | --- | --- | --- |
| Introduction |  |  |  |
| A urinary tract infection, or UTI, is an infection in any part of your urinary system. This may include your bladder, urethra, ureters, and/or kidneys. | A urinary tract infection, or UTI, is an infection in any part of your urinary system. This may include your bladder, urethra, ureters, and/or kidneys. Some people may experience **episodes or flares** of UTI symptoms, with no symptoms in between, while some people may experience UTI symptoms that feel **continuous and do not fully subside**. This questionnaire asks about your experience of UTI symptoms and pain or discomfort. | Clarity: Vague,  Missing info | C08: “I personally use like, use language of like flare. Kind of the community of UTI, UTI community, we might talk about flaring symptoms more, but a temporary increase in symptoms… I knew what I thought you meant by episode”  C11: “[Question A1] does make me feel a bit insecure… am I right candidate for this survey, because is the survey about recurrent UTI? … I cannot say that my UTI symptoms, like they’re not continuous … might be helpful, or even in the part where you say, a urinary tract infection is in your urinary system, maybe also adding an extra passage just to indicate that this survey will … like, if you don’t experience continuous symptoms, don’t worry, kind of thing” |
| Section A |  |  |  |
| The following questions are about how often you experience UTIs. Please consider UTIs that may or may not have been medically diagnosed. | The following questions are about how often you experience UTI symptoms. Please consider UTIs that may or may not have been medically diagnosed. | Conflicting instructions | [related to updates to A1] |
| A1. Do you feel you have had one non-stop UTI with continuous symptoms for the **past 3 months or more**? | Have you had UTI symptoms that feel continuous and do not fully subside for the **past 3 months or more**? | Clarity: Vague | C14: “Do you feel you have one had one non-stop UTI? My answer would be yes, I suspect I may have. But then it says with continuous symptoms, which I don’t have, so I don’t know … I do have some form of infection that has been continuously present, but the symptoms have not been continuous”  C07: “the non-stop, that was the one element that made me think and I was like, Well, yeah, it was pretty much non-stop. I got one in March, and April. May was a bit better, but then June, I had another one” |
| If you selected “Yes”, please skip to section B. If you selected “No”, please continue with the rest of Section A. | − | − | − |
| The term “**episode**” is used here to describe a temporary increase in UTI symptoms before they return to a level that is normal for you. | − | − | − |
| A2. Approximately how many episodes of UTI symptoms have you had in the **past 6 months**? | − | − | − |
| A3. Approximately how many episodes of UTI symptoms have you had in the **past 12 months**? | − | − | − |

*Note.* Code(s) from the Question Appraisal System – 99.(26) Items are written as they are presented in the measure (i.e., with underlined or bold phrasing). Updated items were taken forward for testing in the second phase of patient cognitive interviews.

Hyphens (−) indicate that no changes were made to the corresponding item.

| Instruction/item tested at Phase 1 | Updated instruction/item | Code(s) | Quotation(s) |
| --- | --- | --- | --- |
| Section B |  |  |  |
| The following questions are about your **UTI symptoms other than pain.** | The following questions are about your **UTI symptoms other than pain or discomfort.** | Clarity: Vague, Inappropriate assumptions | C01: “to me, the discomfort felt from a UTI is not pain. It’s really extreme discomfort as opposed to pain. … For some people pain, they might experience a lot of pain. For me, it’s more discomfort. Extreme discomfort”  C16: “we’re talking about discomfort. We’re not talking about pain … I think that's good that you've said more discomfort in there. When you go to doctor's office, they always ask you for pain scale. And I always resent that because it's not painful. I hate to be a liar and say I'm in pain and they always ask for a pain scale” |
| Please indicate whether you had any of the following symptoms in the PAST 24 HOURS, and if so, how SEVERE they were: | Please indicate whether you have experienced any of the following symptoms related to UTI in PAST 24 HOURS, and if so, how SEVERE they were: | Clarity: Vague | [No specific quotation; updated for consistency to match phrasing in other instructions] |
| B1. Needing to urinate more urgently or more suddenly than normal. | Order change: frequency to come before urgency | Clarity: Vague | C07: “Ah, sorry. Urgently, frequently … I responded frequently, not urgently … they’re similar to me and the first in my mind is always the frequency”  C15: “maybe the other way around would be better … do you need to go more frequently, yes, but then sometimes you need to rush off and do it … the second question is kind of backtracking a little on the first question” |
| B2. Needing to urinate more frequently than normal. | Order change: frequency to come before urgency | Clarity: Vague | [relates to updates for B1] |
| B3. Unintentionally passing or leaking urine. | − | − | − |
| B4. Feeling as though you are unable to completely empty your bladder. | − | − | − |
| B5. Feeling as though you have the urge to urinate despite having just urinated. | − | − | − |
| B6. Urine with an unusually strong or unpleasant smell. | − | − | − |
| B7. Visible blood in your urine. | − | − | − |
| B8. Cloudy urine. | − | − | − |
| B9. Debris or floating particles in your urine. | − | − | − |
| B10. Fever (feeling hot with a temperature higher than 38°C or 100.4°F). | − | − | − |
| B11. Chills (feeling cold and shivery). | − | − | − |
| Scale: 0 = not present; 1 = very mild; 10 = worst imaginable. | 0 = not present; 1 = very mild; 10 = extremely severe | Responses: Mismatch | C02: “It’s quite hard to differentiate what would be like the worst imaginable, I think”  C05: “Worst imaginable was more kind of, I kind of have different definitions of like worst imaginable”  C08: “Worst imaginable is a weird description of needing to urinate more urgently or more suddenly than normal … I kind of like, I associate that with like pain” |
| NEW ITEM | Feeling generally unwell. | − | C06: “In the beginning, feeling really sick, unwell, I knew I had an infection … It’s a bit like I’m getting the flu, you know. Just feeling, Oh, hot and cold”  C14: “For me, just the feeling of being so generally unwell … you feel so unwell all over”  C15: “Maybe a bit of … feeling unwell. [I would separate it from fever because] I did check my temperature, it was normal. Although I have some feeling, I’m not like okay” |
| NEW ITEM | Feeling fatigued or low in energy. | − | C04: “Kind of makes me more lethargic”  C06: “Lethargic, really tired. Low energy”  C14: “I thought, Oh, I’m so tired. I guess tiredness and fatigue wasn’t mentioned in there … I was feeling very tired” |

*Note.* Code(s) from the Question Appraisal System – 99.(26) Items are written as they are presented in the measure (i.e., with underlined or bold phrasing). Updated items were taken forward for testing in the second phase of patient cognitive interviews.

Hyphens (−) indicate that no changes were made to the corresponding item.

| Instruction/item tested at Phase 1 | Updated instruction/item | Code(s) | Quotation |
| --- | --- | --- | --- |
| Section C |  |  |  |
| The following questions are about any pain or discomfort related to your UTI(s). | The following questions are about any pain or discomfort in your lower abdomen, genitals and/or bladder, related to your UTI(s). | Complicated instructions,  Double-barrelled | [related to C1-C3, bringing pain locations into instruction vs. individual questions]  C10: “Maybe and/or would be better because, when looking at it, I feel like it’s all three together. So and/or, then I could just kind of filter that through and say, Okay, you know, the bladder pain in the past 24 hours has been about two to three” |
| C1. When you are urinating, how has your lower abdominal, genital or bladder pain been **on average over the past 24 hours**? | When you are urinating, how has your pain or discomfort been on average over the past 24 hours? | Complicated instructions,  Double-barrelled, Inappropriate assumptions | C10: “It’s a long question, and I guess it depends. It’s, this one’s a little bit tough because… It’s kind of asking three questions in one … You don’t always feel all of them at once … when I void, there’s pain more in the bladder or urethra, but not really the abdomen, so how would I rate this?” |
| C2. When you are not urinating, how has your lower abdominal, genital or bladder pain been **on average over the past 24 hours**? | When you are not urinating, how has your pain or discomfort been on average over the past 24 hours? | Complicated instructions,  Double-barrelled, Inappropriate assumptions | [relates to updates for C1] |
| C3. What is your level of lower abdominal, genital or bladder pain **right now**? | What is your level of pain or discomfort right now? | Complicated instructions,  Double-barrelled, Inappropriate assumptions | [relates to updates for C1] |
| Please indicate whether you have experienced any of the following symptoms related to UTI in the PAST 24 HOURS, and if so, how SEVERE they were: | − | − | − |
| C4. Pain or burning sensation when you are urinating. | − | − | − |
| C5. Pain or burning sensation after urinating. | Pain or burning sensation within the 30 minutes  after urinating. | Clarity: Vague | C03: “The only thing I’m thinking there is how soon after urinating? Does it mean immediately?”  C02: “Maybe not straight after but maybe about 10, 15 minutes afterwards. Yeah, because usually actually straight afterwards, it’s actually not so bad immediately afterwards, and then 15 minutes I start to feel all that burning again”  C12: “the pain for me often lingers” |
| C6. Pain or discomfort around the urethra when you are not urinating. | − | − | − |
| C7. Pain or discomfort in your pelvis or lower tummy/abdomen. | Pain or discomfort in your pelvis or lower tummy/abdomen (including bladder pressure). | Clarity: Vague | C13: “Bladder pressure … that’s a big symptom of mine is the pressure”  C14: “the only other feature of it is the pain and pressure feeling in the abdomen, in the bladder” |
| C8. Pain or discomfort in your side/flank. | Order change: lower back pain to come before side pain | Clarity: Vague | C03: “So I always think about flank as being the back, sort of side at the back”  C06: “I think my, my understanding of flank is the side. I, what I was describing [for C8] was in my lower back. Yeah, so I haven’t had pain in the side or the flank. I have had pain in the back, lower back” |
| C9. Pain or discomfort in your lower back. | Order change: lower back pain to come before side pain | Clarity: Vague | [related to C8] |
| Scale: 0 = not present; 1 = very mild; 10 = worst imaginable pain | 0 = not present; 1 = very mild; 10 = extremely severe | Responses: Mismatch | C02: “It’s quite hard to differentiate what would be like the worst imaginable, I think”  C05: “Worst imaginable was more kind of, I kind of have different definitions of like worst imaginable”  [Match Section B] |
| NEW ITEM | Pain or discomfort radiating down into your legs. | - | C09: “I got quite a lot of nerve pain in my left leg … nervy pain in my inner thigh, and sometimes it goes right down to the sole of my foot … radiating, yeah … but when I go to the toilet, I know that will go”  C17: “I don’t really have pain anywhere but in my urethra and in my legs” |

*Note.* Code(s) from the Question Appraisal System – 99.(26) Items are written as they are presented in the measure (i.e., with underlined or bold phrasing). Updated items were taken forward for testing in the second phase of patient cognitive interviews.

Hyphens (−) indicate that no changes were made to the corresponding item.

| Instruction/item tested at Phase 1 | Updated instruction/item | Code(s) | Quotation |
| --- | --- | --- | --- |
| Section D |  |  |  |
| Finally, please indicate whether you: | Please indicate whether you: | − | − |
| D1. Are on your period/menstruating or experiencing other vaginal bleeding (e.g. spotting, perimenopausal bleeding). | Experiencing vaginal bleeding (e.g. period/menstruation, spotting, perimenopausal bleeding).  Order change: to come after D5-7 (universal questions). | Clarity: Vague | C06: “I have [experienced vaginal bleeding] with intercourse … I think that’s what threw me off because it seems very period-related, and as I’m not menstruating anymore and I’m post-menopause, it doesn’t, it’s not applicable, really”  C07: “It’s very easy because you include spotting, because that was my question before reading the whole question. So yeah, it’s great that you included that”  C15 (male): “That would be more applicable to a lady or a woman, would it not? I can’t, I can’t answer those questions at all … I would just skip that whole set of questions instead of starting to read them and going, Well, that’s not for me” |
| D2. Are experiencing premenstrual symptoms (e.g. tummy pain or cramps). | Order change: to come after D5-7 (universal questions). | Other | [as above D1 quotation from participant C15] |
| D3. Are experiencing menopausal symptoms (e.g. vaginal dryness or pain, hot flushes, night sweats). | Are experiencing menopausal or perimenopausal symptoms (e.g. vaginal dryness or pain, hot flushes, night sweats).  Order change: to come after D5-7 (universal questions). | Clarity: Vague | C13: “Well, I’m perimenopausal at the moment, so I do, at the moment get a bit of vaginal pain”  [as above D1 quotation from participant C15] |
| D4. Are pregnant. | Order change: to come first of non-universal questions, which will come after D5-7 (universal questions). | Other | [as above D1 quotation from participant C15] |
| D5. Have diabetes (of any type). | Order change: D5-D7 to come first (universal questions) | Other | [as above D1 quotation from participant C15] |
| D6. Use intermittent or indwelling catheterisation to drain your bladder. | Have used any type of catheterisation to drain your bladder in the past week.  + Order change | Clarity: Vague, Other | C15: “I’m not sure what indwelling means … as somebody who’s unfamiliar with it, don’t, it’s hard to follow the question but I kind of understand it”  [as above D1 quotation from participant C15] |
| D7. Have experienced constipation in the past 24 hours. | Order change | Other | [as above D1 quotation from participant C15] |
| Responses: Yes; No; Not applicable | Yes; No | Responses: Mismatch | C02: “[to D2] I would say no, but that's because I'm actually in an induced menopause right now. I don't know if that would either be no or not applicable. I don't know”  C03: “So I'm assuming that will be not applicable, because I, I am past the menopause. But I'm not having any symptoms. So I'm a little bit confused about, whereas the first ones I've assumed where it's saying are you on your period or menstruating, well, I wouldn't be because that's not applicable because I don't anymore. So I'm assuming that would be not applicable”  C15: “I mean, D5 to D7 is just yes or no. How's it not applicable? You either have or you haven't. Like, I can't possibly have constipation, because I'm sort of some weird creature” |
| NEW INSTRUCTION | [To go before originally labelled D1-D4]  The following questions are specific to female biological sex. If applicable, please indicate whether you are currently: | Clarity: Vague, Conflicting Instructions | [as above D1 quotation from participant C15] |

*Note.* Code(s) from the Question Appraisal System – 99.(26) Items are written as they are presented in the measure (i.e., with underlined or bold phrasing). Updated items were taken forward for testing in the second phase of patient cognitive interviews.

Hyphens (−) indicate that no changes were made to the corresponding item.

| Original instruction/item | Updated instruction/item | Code(s) | Quotation |
| --- | --- | --- | --- |
| NEW SECTION | [New Section B – after symptom frequency and before symptom/pain severity questions]  The following questions are about any change in your UTI symptoms. | − | C08: “I guess I'm thinking if I was given this at the Doctors, right? And they were saying, Okay, you've come in, we're gonna give you dipstick, [C08 name], can you complete this as well? I'd kind of want to also talk about like, the whole episodic symptoms. Because what if it hadn't been that bad in these 24 hours? But like, two days ago, it was agonizing or like, I kind of wonder if there's scope to capture the symptoms through your whole current episode, not just the 24 hours that you're having to the doctors in … I like [the idea of an improved/worsened scale], I think that gives a greater indication of like, the whole episode and where you are in that or Yeah, exactly, have things got better or worse over the last 24 hours?”  C18: “Because 24 hours is sometimes, Okay, you’ve got a good day or you’ve got a really bad day” |
|  | B1. Please consider how you typically experience UTI symptoms.  To what extent have your UTI symptoms over the PAST 24 HOURS been better or worse than your typical experience? |  |  |
|  | Scale: -5 = very much worse; 0 = no change; +5 = very much better |  |  |

*Note.* Code(s) from the Question Appraisal System – 99.(26) Items are written as they are presented in the measure (i.e., with underlined or bold phrasing). Updated items were taken forward for testing in the second phase of patient cognitive interviews.

Hyphens (−) indicate that no changes were made to the corresponding item.

Qualitative feedback and refinements made after the second phase of patient cognitive interviewing

| Instruction/item tested at Phase 2 | Updated instruction/item | Code(s) | Quotation |
| --- | --- | --- | --- |
| Introduction |  |  |  |
| A urinary tract infection, or UTI, is an infection in any part of your urinary system. This may include your bladder, urethra, ureters, and/or kidneys. Some people may experience **episodes or flares** of UTI symptoms, with no symptoms in between, while some people may experience UTI symptoms that feel **continuous and do not fully subside**. This questionnaire asks about your experience of UTI symptoms and pain or discomfort. | A urinary tract infection, or UTI, is an infection in any part of your urinary system. This may include your bladder, urethra, ureters, and/or kidneys. Some people may experience **episodes of UTI symptoms with no symptoms in between**, while some people may experience **UTI symptoms that feel continuous and do not fully subside**. This questionnaire asks about your experience of UTI symptoms and pain or discomfort. | Conflicting or inaccurate instructions | C19: “Yeah, I mean, episodes or flares, I mean, flare ups, I think is something I would, you know, is a term that I would better identify with … [to A1] Yes … I’m interpreting that as what I call my background symptoms”  C21: “I wouldn't really have known about this, but I joined the group, this embedded UTI group, and this is the things that they use that they speak, I will often see like flare, which I wouldn't have known that was what that was” |
| The following questions are about how often you experience UTI symptoms. Please consider UTIs that may or may not have been medically diagnosed. | − | − | − |
| A1. Have you had UTI symptoms that feel continuous and do not fully subside for the **past 3 months or more**? | Have you had UTI symptoms that feel continuous and do not fully subside for **at least the past 3 months or more**? | Clarity: Vague,  Double-barrelled | C24: “I have had UTI symptoms in the past three months, but they are not continuous. And I was able to fully cure them at each episode. So is that a yes or no? Because it's not, that they... Yes, I've had them in the past three months, but I was able to get rid of them all, each individual time, and it was not continuous”  C25: “the question to me, was not 100% clear on, Have I had continuous UTI symptoms for the past three months? Or have I had like, continuous over the past three months? Or have I had UTI symptoms that were continuous within the past three months anytime like within the last three months? … [At least the past 3 months would be better] if your intention is that if it’s been continued, they symptoms have not gone away for the past three months” |
| If you selected “Yes”, please skip to section B. If you selected “No”, please continue with the rest of Section A. | − | − | − |
| A2. Approximately how many episodes of UTI symptoms have you had in the **past 6 months**? | − | − | − |
| A3. Approximately how many episodes of UTI symptoms have you had in the **past 12 months**? | − | − | − |

*Note.* Code(s) from the Question Appraisal System – 99.(26) Items are written as they are presented in the measure (i.e., with underlined or bold phrasing).

Hyphens (−) indicate that no changes were made to the corresponding item.

| Instruction/item tested at Phase 2 | Updated instruction/item | Code(s) | Quotation |
| --- | --- | --- | --- |
| Section B |  |  |  |
| The following questions are about any change in your UTI symptoms. | − | − | − |
| Please consider how you typically experience UTI symptoms.  To what extent have your UTI symptoms over the PAST 24 HOURS been better or worse than your typical experience? | − | − | − |
| Scale: -5 = very much worse; 0 = no change; +5 = very much better | − | − | − |

*Note.* Code(s) from the Question Appraisal System – 99.(26) Items are written as they are presented in the measure (i.e., with underlined or bold phrasing).

Hyphens (−) indicate that no changes were made to the corresponding item.

| Instruction/item tested at Phase 2 | Updated instruction/item | Code(s) | Quotation |
| --- | --- | --- | --- |
| Section C |  |  |  |
| The following questions are about your UTI symptoms other than pain or discomfort. | − | − | − |
| Please indicate whether you have experienced any of the following symptoms related to UTI in the PAST 24 HOURS, and if so, how SEVERE they were: | − | − | − |
| C1. Needing to urinate more frequently than normal. | − | − | − |
| C2. Needing to urinate more urgently or more suddenly than normal. | − | − | − |
| C3. Unintentionally passing or leaking urine. | − | − | − |
| C4. Feeling as though you are unable to completely empty your bladder. | − | − | − |
| C5. Feeling as though you have the urge to urinate despite having just urinated. | − | − | − |
| C6. Urine with an unusually strong or unpleasant smell. | − | − | − |
| C7. Visible blood in your urine. | − | − | − |
| C8. Cloudy urine. | − | − | − |
| C9. Debris or floating particles in your urine. | − | − | − |
| C10. Feeling generally unwell. | − | − | − |
| C11. Feeling fatigued or low in energy. | ITEM REMOVED | Clarity: Vague,  Other | C25: “Now that one for me is a very difficult one to distinguish to a UTI because the fatigue is very much part of my cancer as well. And, and I feel that a lot. So, and I mean, I do feel it even more so when I have a UTI. But that one is even hard. That one's hard for me to distinguish. I mean, I would say right now is probably at about a four, but I wouldn't know if 100% if it's related to a UTI”  C24: “[for C10] I guess that's when the fatigue and the low energy comes in. So I'm gonna say generally unwell, I'd give that a four”  C27: “[for C10] I mean tired I get, I get, I have a lot of, which is the next one, I see … I guess the tiredness stems from the fact that I don’t sleep through the night like I used to … that’s a tough one”  C28: “[for C10] I feel tired when I have a UTI, and tiredness is probably in the scale up to four … [C11] around four” |
| C12. Fever (feeling hot with a temperature higher than 38°C or 100.4°F). | − | − | − |
| C13. Chills (feeling cold and shivery). | − | − | − |
| Scale: 0 = not present; 1 = very mild; 10 = extremely severe | − | − | − |

*Note.* Code(s) from the Question Appraisal System – 99.(26) Items are written as they are presented in the measure (i.e., with underlined or bold phrasing).

Hyphens (−) indicate that no changes were made to the corresponding item.

| Instruction/item tested at Phase 2 | Updated instruction/item | Code(s) | Quotation |
| --- | --- | --- | --- |
| Section D |  |  |  |
| The following questions are about any pain or discomfort in your lower abdomen, genitals and/or bladder, related to your UTI(s). | − | − | − |
| D1. When you are urinating, how has your pain or discomfort been **on average over the past 24 hours**? | − | − | − |
| D2. When you are not urinating, how has your pain or discomfort been **on average over the past 24 hours**? | − | − | − |
| D3. What is your level of pain or discomfort **right now**? | − | − | − |
| Please indicate whether you have experienced any of the following symptoms related to UTI in the PAST 24 HOURS, and if so, how SEVERE they were: | − | − | − |
| D4. Pain or burning sensation when you are urinating. | − | − | − |
| D5. Pain or burning sensation after urinating. | − | − | − |
| D6. Pain or discomfort around the urethra when you are not urinating. | − | − | − |
| D7. Pain or discomfort in your pelvis or lower tummy/abdomen (including bladder pressure). | − | − | − |
| D8. Pain or discomfort in your lower back. | − | − | − |
| D9. Pain or discomfort in your side/flank. | − | − | − |
| D10. Pain or discomfort radiating down into your legs. | − | − | − |
| Scale: 0 = not present; 1 = very mild; 10 = extremely severe | − | − | − |

*Note.* Code(s) from the Question Appraisal System – 99.(26) Items are written as they are presented in the measure (i.e., with underlined or bold phrasing).

Hyphens (−) indicate that no changes were made to the corresponding item.

| Instruction/item tested at Phase 2 | Updated instruction/item | Code(s) | Quotation |
| --- | --- | --- | --- |
| Section E |  |  |  |
| Please indicate whether you: | − | − | − |
| E1. Have diabetes (of any type). | − | − | − |
| E2. Have used any type of catheterisation to drain your bladder in the past week. | − | − | − |
| E3. Have experienced constipation in the past 24 hours. | − | − | − |
| The following questions are specific to female biological sex. If applicable, please indicate whether you are currently: | The following questions are specific to females and people assigned female at birth. If applicable, please indicate whether you are currently: | Clarity: Vague | C19: “It's an odd phrase, female biological sex. I wonder whether the following questions are specific to female biology. Rather than, you know, I read that and sort of go, are they going to ask me how often I'm having sex and that's not what you're asking at all. That's just wording. Or the following questions are specific to females”  C20: “Some people might say, Well, why doesn't it just say women, but I think in this day and age, female biological sex won't cover everyone”  C21: “I have a real problem using the word sex here but that's because over the years we've got ourselves indoctrinated into using gender and sex just means intercourse”  C28: “I haven't had sex for [the] last two years, so It doesn't matter” |
| E4. Pregnant. | − | − | − |
| E5. Experiencing vaginal bleeding (e.g. period/menstruation, spotting, perimenopausal bleeding). | − | − | − |
| E6. Experiencing premenstrual symptoms (e.g. tummy pain or cramps). | − | − | − |
| E7. Experiencing menopausal or perimenopausal symptoms (e.g. vaginal dryness or pain, hot flushes, night sweats). | − | − | − |
| Responses: Yes; No | − | − | − |

*Note.* Code(s) from the Question Appraisal System – 99.(26) Items are written as they are presented in the measure (i.e., with underlined or bold phrasing).

Hyphens (−) indicate that no changes were made to the corresponding item.
